# Strong selection for behavioural resilience in Australian stock working dogs identified by selective sweep analysis

**DOI:** 10.1186/s40575-015-0017-6

**Published:** 2015-05-07

**Authors:** Elizabeth R Arnott, Lincoln Peek, Jonathan B Early, Annie Y H Pan, Bianca Haase, Tracy Chew, Paul D McGreevy, Claire M Wade

**Affiliations:** Faculty of Veterinary Science, University of Sydney, Camperdown, NSW 2006 Australia

**Keywords:** Kelpie, Behaviour, Selective sweep

## Abstract

**Background:**

Working dog handlers and breeders have strong opinions on characteristics that are desirable in the breeds that they use to handle stock. Most of these characteristics are related to conformation or behaviour. This study explored whether the genetics underlying desirable working behaviour traits might be identified by selective sweep analysis; a method that identifies long regions of strong homozygosity combined with allelic divergence from a comparison group. For this analysis, we compared genomic haplotype architecture in two breeds derived from common founder stock but subjected to divergent selective pressures. The breeds studied were the Australian Kelpie, which is registered with the Australian National Kennel Council, and the Australian Working Kelpie, which is registered with the Working Kelpie Council.

**Results:**

A selective sweep spanning 3 megabases on chromosome 3 was identified in the Australian Working Kelpie. This region is the location of genes related to fear-memory formation and pain perception.

Selective sweep loci of similar magnitude were observed in the Australian Kelpie. On chromosome 8 is a locus which may be related to behavioural excitability and on chromosome 30 is a smaller locus which most likely is related to morphology.

**Conclusions:**

Active working stock dogs of the Australian Working Kelpie breed have been bred primarily for gene loci influencing pain perception and fear memory formation. By contrast Australian Kelpies are commonly maintained in urban environments where these characteristics are not required and have been affected by selection for conformation and coat colour. The identified loci may aid in the identification of superior working dogs.

**Electronic supplementary material:**

The online version of this article (doi:10.1186/s40575-015-0017-6) contains supplementary material, which is available to authorized users.

## Lay summary

The term ‘selective sweep’ is used in the study of genetics to describe a reduction or loss of DNA sequence variation in regions of the genome of species, breeds or cultivars. This can occur as a consequence of strong selective pressure (positive evolutionary selection) due to a highly desirable DNA mutation in such regions which convey a survival advantage (positive evolutionary natural selection). Alternatively it can be artificially driven by man in other species through manipulating selection with eugenic breeding programmes. A selective sweep analysis can be very helpful in both determining the close genetic relationship of individuals and groups within a species, and also in some cases can be useful in identifying gene variants that cause disease.

In this study of Australian Kelpies we have applied a fresh approach to selective sweep analysis that interrogates a breed ‘split’ so as to learn more about the ‘external characteristics’ (often called the phenotype) that are regarded as desirable in two very different cohorts of dogs that share a common breed origin. One group of dogs is intensively selected for its ability to work with livestock, while the other group is bred for conformation and companionship – usually in an urban setting. This study suggests that “working Kelpies” have a strong genomic selection signal in a region of their genome that contains genes predominantly concerned with resilience traits such as the ability to sense pain and form memories associated with fear. The “nonworking” Kelpie group have selection signals that are most likely related to body shape and size (conformation traits).

A selective sweep is the reduction or elimination of variation among the nucleotides in neighboring DNA of a mutation as the result of recent and strong positive natural or artificial selection.

## Background

Selection, both natural and artificial, can be very powerful in shaping phenotype and, as widely described in the literature, this is broadly demonstrated in the morphologies and behaviours of domestic dogs. Previous studies have assessed selective sweep by comparison of disparate dog breeds [1-6]. In this work, we concentrate on comparing two closely related breeds that have been derived from a common ancestor but then bred for different purposes. By doing so, we expect to be able to more easily identify regions that underpin the observable behavioural and physical differences between the breeds.

Where selection is based upon a common breeding goal, selective progress in breed improvement is enhanced; a strategy widely used in the production animal industries. Selection in dog populations is assisted by the birth of progeny in litters that may number as high as 15 individuals but are more commonly in the range of 5–7 offspring. The prolificacy enables breeders to exert considerable selection pressure within a family, albeit with a demonstrably low correlation between pup behavioural assessment and adult working success [7]. A relatively large amount of selection pressure in dog breeding occurs at a young age (approximately 8 weeks) when the breeder elects either to keep a pup potentially for breeding or to sell it. A second stage of selection occurs when the adult is identified as being suitable for breeding.

**Figure 1:**
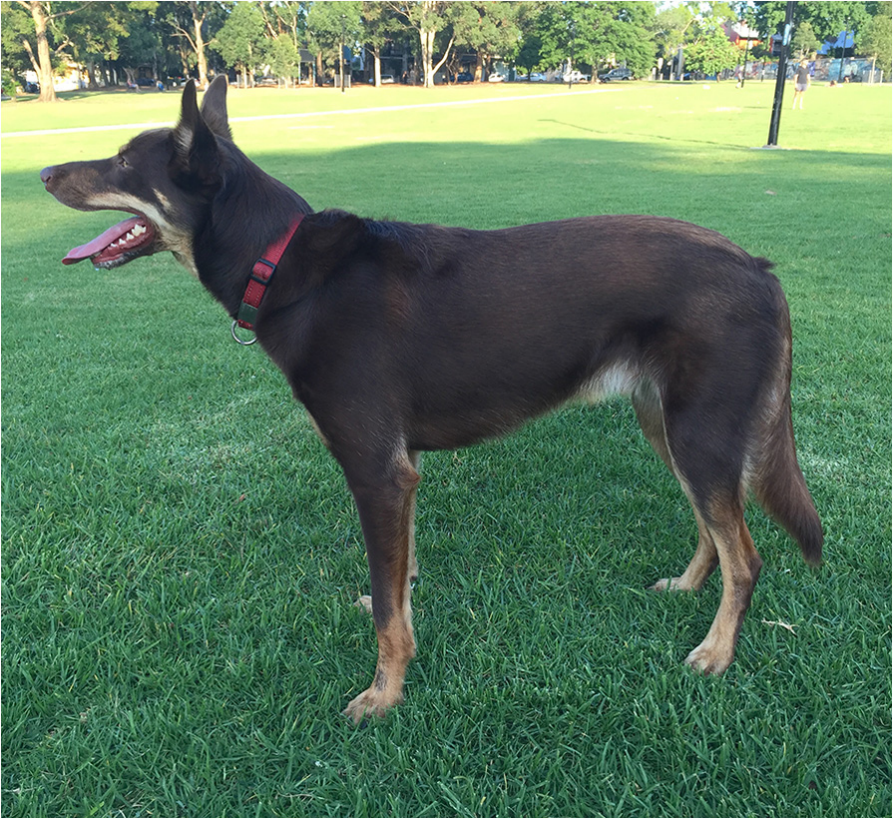
Australian Working Kelpie (photo credit: Jonathan Early).

The Australian Working Kelpie (AWK) breed represents dogs registered with the Working Kelpie Council (WKC) and is the product of more than a century of breeding by Australian sheep and cattle farmers who have focused on stock working ability (Figure 1). The WKC describe an early focus their breeding strategy, “*Selection from the beginning was for a sheepdog that could cope with the conditions. This having been obtained with the early crossings the features* (sic) *has been rigorously retained....... revels in hard going. Established specially for local conditions he is able to muster huge areas under extreme conditions, often having to do without water for hours on end*” [8]. The requirements for a dog working stock in a yard or during transport are recognised in the working dog breeder community as being quite different from those required to herd stock in a large area with often difficult terrain, or from the requirements of a breeder who is interested in competitive sheepdog trials [9-12]. However, we expect that the basic ability to effectively work stock might have a common basis and in this study we are interested to explore whether we might uncover this through genomic analysis.

**Figure 2:**
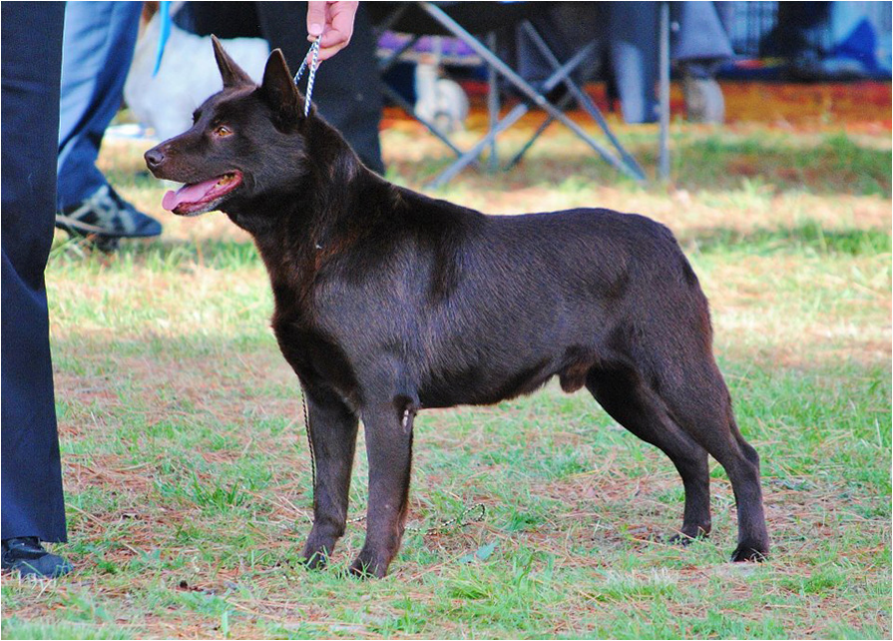
Australian Kelpie (photo credit: Jenny Bayliss Photography).

Breeders of the Australian Kelpie (AK) representing dogs registered with the Australian National Kennel Council (ANKC) sell pups primarily into companion homes and are more likely to engage in activities such as dog showing and other competitive dog sports that are not related to stock work (such as obedience and agility) (Figure 2). The AK was first exhibited in 1908 and the breed standard was adopted in 1963 [13]. Both breeds are originally derived from a bitch known as Kings Kelpie that won the first sheepdog trial in New South Wales [13]. In the early years of the breed many of the dogs were black and tan but solid black dogs were derived from a male known as Moss who was later bred with Kings Kelpie and produced a popular sire known as Barb (named for a black racehorse) [13].

Methodologies for the assessment of selective sweep have been explored extensively in the literature [6,14,15]. For the current study, we elected to use a simple analysis of extended homozygosity over one megabase genomic windows combined with a long region of heterogeneous allele frequency (F_ST_) [16] relative to the alternate population with the goal of identifying genomic regions that have responded best to the strong selective forces applied by breeders.

## Results and discussion

The results of the homozygosity analysis are shown in Table 1 for the Australian Working Kelpie and the Australian Kelpie. A single major locus of sweep spanning 4 Mb was identified for the Australian Working Kelpie. The bounds of this sweep were on canine chromosome 3 spanning from 26 megabases (Mb) to 30 Mb on the canine reference assembly CanFam3.0 [17,18].

**Table 1:** **Regions identified in the primary analysis for differential selective sweep including 12 Australian Kelpie and 12 Australian Working Kelpie dogs that were then assessed for validation in a further 10 AK and 28 AWK individuals**

				**Primary**			**Validation**		
	**Chromosome**	**Position (1 Mb bin) canfam 3.0**	**Markers in window**	**maf** ^**£**^ **AWK** ^**†**^	**maf AK** ^**‡**^	**maf absolute difference**	**maf AWK** ^**†**^	**maf AK** ^**‡**^	**maf absolute difference**
AWK	3	26000000	64	0.012	0.176	0.164	0.027*	0.187	0.16
	3	27000000	79	0.012	0.194	0.181	0.031*	0.187	0.156
	3	29000000	70	0.009	0.178	0.169	0.015*	0.151	0.135
	3	30000000	66	0.008	0.161	0.153	0.082	0.178	0.096
	10	47000000	72	0.048	0.169	0.121	0.101	0.173	0.071
	11	2000000	40	0.016	0.184	0.169	0.022	0	0.022
	11	17000000	83	0.05	0.179	0.13	0.148	0.182	0.034
	11	36000000	71	0.041	0.186	0.144	0.062	0.118	0.056
	15	46000000	72	0.031	0.195	0.164	0.106	0.21	0.104
	24	46000000	88	0.025	0.16	0.136	0.052	0.153	0.1
	30	1000000	82	0.011	0.13	0.119	0.014	0.085	0.071
	30	5000000	41	0.046	0.162	0.116	0.092	0.022	0.069
	39	102000000	47	0.026	0.153	0.127	0.213	0.23	0.017
AK	8	64000000	81	0.166	0.049	0.118	0.189	0.049*	0.141
	8	65000000	75	0.165	0.042	0.123	0.221	0.040*	0.182
	8	67000000	71	0.13	0.008	0.122	0.131	0.004*	0.126
	30	23000000	87	0.193	0.029	0.164	0.178	0.016*	0.162
	39	16000000	42	0.208	0.048	0.161	0.057	0.075	0.018

^†^Australian Working Kelpie.

^‡^Australian Kelpie.

^£^minor allele frequency (maf).

*supported in expanded analysis.

Two loci were identified for the Australian Kelpie. The first is on chromosome 8 and spans 4 Mb from 64 to 68 Mb with the strongest signal at 67 Mb. The second is on chromosome 30 and is supported in the wider data for a single window at 23 Mb. Adjoining windows spanning an area of 21 Mb to 25 Mb maintained mean minor allele frequencies of lower than 0.07 in the larger data cohort.

Positional candidate genes for behavioural and morphological traits are shown in Table 2 for the three supported sweep regions.

**Table 2:** **Positional candidate genes for behaviour and morphology phenotypes according to the mouse genome browser**

**Positional candidate genes**						
**Canine Locus (Canfam 3.0)**	**Mouse Locus (GRCm38/mm10)**	**Sweep cohort**	**Behaviour**	**Integument/Pigmentation**	**Craniofacial**	**Skeletal**
chr3:26-30 Mb	chr13:91.8-95.7 Mb	AWK^†^	Homer1, Arsb, Lhfpl2, Ap3b1,Crhbp, F2rl1	Msh3, Homer1, Ap3b1,F2rl1	Arsb, Ap3b1	Arsb, F2rl1
chr8:64-68 Mb	chr12:105-109 Mb	AK^‡^	Dicer1, Bdkrb2,Bdkrb1, Bcl11b	Dicer1, Bdkrb2,Bdkrb1, Bcl11b	Dicer1, Ak7, Bcl11b	Dicer1, Bdkrb2, Bcl11b
chr30:23-24 Mb	chr9:70.5-71.5 Mb	AK^‡^	-	-	Adam10, Aldh1a2	Adam10, Aldh1a2

^†^Australian Working Kelpie.

^‡^Australian Kelpie.

Both breeds appeared to have a single predominant common haplotype on the X chromosome (Additional file 1: Table S1). This is not surprising as both groups in our data were founded on a single female that had offered exceptional working ability [8]. One additional locus of sweep for the AK was excluded from the reported results due to low density of markers in the window (at chr16:59000000) but is nonetheless observably supported in the dog phenotypes. The Australian Kelpie is frequently self-coloured brown or black (although the brown may be reported as either red or chocolate by breeders, depending on the hue). In contrast, Australian Working Kelpies may be the same colours as those reported for the AK, but most frequently exhibit tan markings (commonly referred to as black and tan or red and tan). One window of selective sweep for the AK (chr16:59000000) sits directly on the K-locus (*DEFB103*) [19] that affects the expression of pheomelanin versus eumelanin in the dog. No sweep was observed in either breed for the agouti locus that has been previously described as the major locus underlying the black and tan coat colour phenotype [20,21].

It is not the first time that the locus on chromosome 3 for the AWK has been identified in research on this breed. In an analysis to identify a putatively recessive locus underpinning an inherited disorder in Kelpies, Shearman et al. [22] identified this exact same region as a region of extended homozygosity in samples affected by cerebellar ataxia. We predict that the earlier disease association study had included a mixture of AK and AWK in the control cohort and that the signal identified is based on cohort stratification. While some dogs in the current population participated in the study of cerebellar ataxia, our analysis excluded all case samples from the data and doing so clearly reveals that homozygosity in this region is a characteristic of all AWK.

Presuming that the region in this sweep is affected by strong selection for working success, the most interesting of the positional candidate genes on chromosome 3 is the *HOMER1* gene. This gene is associated with fear memory formation and pain perception (nociception) in the mouse [23-25]. The areas where working stock dogs are employed in Australia have large numbers of environmental hazards. In particular, a large proportion of the groundcovers are of a spiked nature and species such as *Tribulus terrestris* (cat-head burr), *Xanthium spp* (Bathurst burr, Noogoora burr), *Onopordum acanthium* (Scotch thistle), *Alternanthera pungens* (Khaki weed), *Nassella neesiana* (Chilean needle grass) and *Cenchrus spp* (Spiny burrgrass) are common (http://www.weeds.org.au/). In addition, traumatic injuries caused by livestock, fences and vehicles are well documented [26]. A dog that can overcome pain to maintain sufficient focus and continue its work will be a strong asset to the working dog handler. Dogs that are resilient will also have an enhanced chance of working success in the challenging environment of Australian stockwork. Interestingly, an important founder sire for the AWK, a blue dog named “Coil”, is renowned for his exceptional pain tolerance and endurance. Coil won the 1898 Sydney trial achieving a perfect score despite competing with a fractured foreleg [8]. Further research is required to explore whether pain thresholds truly differ between these cohorts of dogs.

For the Australian Kelpie, the major identified sweep locus on chromosome 8 contains genes that relate to both behaviour and morphology but none that stands out as an obvious candidate. The region contains a large gene poor section that has an enriched selection of highly conserved non-coding elements. The gene *BCL11B* (B-Cell Lymphoma/Leukemia 11B) is a transcriptional repressor and one gene, *UNC79* (Protein Unc-79 Homolog) just outside of the swept interval but in the vicinity has been linked with hyperactivity in the mouse [27].

Given the activity of the genes in this region, the driver for the chromosome 30 sweep locus in the AK likely has a morphological, rather than behavioural, basis.

## Conclusions

By focusing on breeds that are derived from a common foundation but then selected for different purposes we have been able to identify a major locus underlying ability of stock dogs, represented by the Australian Working Kelpie, to effectively work in harsh environmental conditions. While stock dog breeders may be selecting primarily for traits such as stock sense and boldness, we reveal that they are favouring breeding from dogs that can focus and continue working despite the presence of environmental hazards and discomforts. This requirement is not needed for dogs accustomed to an urban lifestyle. Australian Kelpies are not usually employed in stock work. They appear to have been subjected to selection that is predominantly based on morphology rather than behaviour.

## Methods

Twelve Australian Working Kelpie (AWK) dogs (registered with the Working Kelpie Council) and twelve Australian Kelpie (AK) dogs (registered with the Australian National Kennel Council) were used in the primary analysis. Ten additional AK (representing data from an unrelated family collected for an unrelated disease study) and 28 additional AWK (collected for unrelated studies) were used to validate the loci identified in the primary analysis. Case samples from the other studies were excluded.

Peripheral blood samples were obtained from six dogs using EDTA blood collection tubes (BD Vacutainer, BD Franklin Lakes, NJ) and extracted using EZ1® DNA Blood Kits (Qiagen, Valencia, CA). The remaining two samples were collected using Oragene ANIMAL OA-400 saliva collection kits (DNA Genotek, Ontario Canada) and extracted following standard kit-issued protocol.

Both primary analysis and validation samples were collected with University of Sydney ethics clearance (N00/10-2012/3/5837 and N00/10-2012/3/5928).

Genotyping was conducted on the Illumina Canine High Density Genotyping array (170,000 markers) by Neogen/Geneseek Nebraska USA.

Results were assessed for minor allele frequency within dog registry cohorts using the --freq option in the Plink software package [28]. Mean minor allele frequencies were binned in one megabase non-overlapping sliding windows across the genome within cohort.

For analysis of the Australian Working Kelpie selective sweep(s), one megabase windows with more than 40 single nucleotide polymorphism (SNP) observations and a mean minor allele frequency of lower than 0.05 were identified and compared with the same window for the Australian Kelpie. Where the absolute difference between minor allele frequencies in the compared groups was greater than the arbitrarily set value of 0.11 the region was selected for validation. Next, windows with a mean minor allele frequency of lower than 0.05 in the Australian Kelpie and an absolute difference in minor allele frequency (>0.11) relative to the Australian Working Kelpie were identified for validation as indicating sweep in the Australian Kelpie.

Finally, both data sets were expanded to include the additional 38 dogs (10 AK and 28 AWK) and the analysis was repeated.

The intervals identified as representing selective sweeps were compared with the conserved syntenic regions on the mouse genome using the mouse genome browser hosted by Mouse Genome Informatics [29]. Genes with relevance for behaviour, integument, craniofacial and skeletal phenotypes were identified using the phenotype utility within the browser.

## Additional file

Additional file 1: Table S1. Relative minor allele frequencies for both Kelpie types (AK and AWK) in one megabase windows across all chromosomes.
