# Supplementary material for: Strong selection for behavioural resilience in Australian stock working dogs identified by selective sweep analysis
Source: Canine Genet Epidemiol. 2015 May 7;2:6. doi: 10.1186/s40575-015-0017-6 (PMC4579362; doi:10.1186/s40575-015-0017-6)
Supplement: Additional file 1: Table S1. — Relative minor allele frequencies for both Kelpie types (AK and AWK) in one megabase windows across all chromosomes. [file 40575_2015_17_MOESM1_ESM.pdf]

Chromosome	Position (Mod)	Count Markers	Mean MAF£ Primary AK‡ (n=12)	Mean MAF£ Validation AK‡ (n=22)	Mean MAF£ Primary AWK† (n=12)	Mean MAF£ Validation AWK† (n=40)	Absolute Difference (Primary)	Absolute Difference (Validation)
1	0	51	0.107	0.114	0.123	0.119	0.015	0.004
1	1000000	81	0.195	0.150	0.131	0.108	0.064	0.042
1	2000000	69	0.108	0.088	0.092	0.102	0.017	0.014
1	3000000	60	0.101	0.092	0.058	0.071	0.043	0.021
1	4000000	73	0.157	0.156	0.096	0.143	0.061	0.013
1	5000000	71	0.086	0.072	0.211	0.162	0.125	0.090
1	6000000	73	0.133	0.104	0.156	0.132	0.023	0.028
1	7000000	51	0.133	0.144	0.154	0.144	0.021	0.000
1	8000000	77	0.183	0.172	0.140	0.148	0.043	0.024
1	9000000	66	0.189	0.179	0.213	0.215	0.024	0.036
1	10000000	54	0.180	0.157	0.162	0.151	0.018	0.007
1	11000000	55	0.156	0.151	0.125	0.144	0.031	0.008
1	12000000	50	0.175	0.132	0.204	0.224	0.029	0.092
1	13000000	66	0.181	0.116	0.169	0.183	0.012	0.067
1	14000000	77	0.159	0.151	0.142	0.160	0.018	0.009
1	15000000	82	0.149	0.175	0.168	0.205	0.020	0.030
1	16000000	71	0.183	0.189	0.130	0.178	0.052	0.011
1	17000000	85	0.179	0.170	0.156	0.169	0.023	0.001
1	18000000	69	0.208	0.170	0.225	0.226	0.017	0.056
1	19000000	80	0.132	0.088	0.097	0.119	0.035	0.031
1	20000000	56	0.148	0.156	0.171	0.179	0.023	0.023
1	21000000	59	0.117	0.106	0.176	0.153	0.059	0.047
1	22000000	80	0.144	0.128	0.160	0.128	0.016	0.000
1	23000000	70	0.153	0.175	0.173	0.228	0.019	0.052
1	24000000	71	0.175	0.170	0.193	0.210	0.018	0.040
1	25000000	62	0.185	0.163	0.129	0.198	0.057	0.035
1	26000000	73	0.172	0.159	0.149	0.144	0.023	0.016
1	27000000	69	0.146	0.137	0.165	0.177	0.020	0.040
1	28000000	70	0.129	0.141	0.211	0.192	0.082	0.052
1	29000000	70	0.144	0.158	0.109	0.129	0.035	0.030
1	30000000	73	0.154	0.152	0.138	0.166	0.016	0.014
1	31000000	66	0.169	0.162	0.175	0.209	0.007	0.047
1	32000000	67	0.147	0.167	0.180	0.212	0.033	0.044
1	33000000	67	0.179	0.192	0.217	0.250	0.038	0.058
1	34000000	81	0.177	0.177	0.141	0.137	0.035	0.040
1	35000000	74	0.141	0.154	0.166	0.163	0.025	0.009
1	36000000	69	0.149	0.181	0.157	0.152	0.008	0.029
1	37000000	75	0.199	0.183	0.179	0.197	0.020	0.015
1	38000000	74	0.155	0.168	0.196	0.195	0.040	0.027
1	39000000	71	0.124	0.133	0.152	0.159	0.028	0.025
1	40000000	56	0.170	0.155	0.209	0.191	0.039	0.036
1	41000000	68	0.209	0.206	0.196	0.214	0.013	0.008
1	42000000	54	0.113	0.112	0.146	0.144	0.032	0.032
1	43000000	64	0.072	0.047	0.107	0.114	0.035	0.066
1	44000000	63	0.198	0.158	0.212	0.220	0.014	0.062
1	45000000	75	0.124	0.091	0.117	0.141	0.007	0.050
1	46000000	71	0.123	0.102	0.105	0.115	0.018	0.013
1	47000000	81	0.173	0.135	0.143	0.149	0.031	0.014
1	48000000	70	0.137	0.111	0.118	0.146	0.019	0.035
1	49000000	78	0.189	0.164	0.109	0.150	0.080	0.015
1	50000000	77	0.148	0.137	0.160	0.195	0.012	0.058
1	51000000	64	0.124	0.132	0.144	0.146	0.020	0.015
1	52000000	67	0.188	0.189	0.159	0.197	0.029	0.008
1	53000000	71	0.190	0.162	0.201	0.215	0.011	0.053
1	54000000	85	0.200	0.160	0.194	0.195	0.007	0.035
1	55000000	87	0.091	0.090	0.084	0.086	0.007	0.004
1	56000000	77	0.173	0.162	0.123	0.126	0.050	0.036
1	57000000	67	0.157	0.132	0.066	0.078	0.091	0.053
1	58000000	71	0.177	0.136	0.124	0.121	0.053	0.015
1	59000000	72	0.171	0.142	0.125	0.094	0.046	0.048
1	60000000	60	0.226	0.164	0.053	0.163	0.173	0.000
1	61000000	59	0.172	0.131	0.095	0.093	0.078	0.038
1	62000000	61	0.206	0.161	0.168	0.197	0.038	0.036
1	63000000	63	0.123	0.111	0.166	0.182	0.043	0.071
1	64000000	64	0.135	0.114	0.188	0.176	0.053	0.062
1	65000000	71	0.092	0.066	0.063	0.071	0.029	0.005
1	66000000	72	0.225	0.195	0.164	0.173	0.061	0.022
1	67000000	70	0.170	0.128	0.149	0.157	0.021	0.029
1	68000000	77	0.162	0.158	0.129	0.154	0.033	0.004
1	69000000	42	0.193	0.259	0.238	0.248	0.045	0.011
1	70000000	47	0.267	0.243	0.249	0.253	0.018	0.010
1	71000000	60	0.084	0.084	0.125	0.130	0.041	0.046
1	72000000	49	0.120	0.104	0.174	0.161	0.054	0.057
1	73000000	56	0.176	0.139	0.161	0.185	0.015	0.045
1	74000000	61	0.169	0.096	0.155	0.155	0.014	0.059
1	75000000	33	0.153	0.136	0.200	0.167	0.047	0.030
1	76000000	59	0.129	0.124	0.150	0.152	0.021	0.028
1	77000000	73	0.120	0.113	0.114	0.100	0.007	0.013
1	78000000	73	0.139	0.117	0.142	0.138	0.002	0.021
1	79000000	69	0.162	0.144	0.149	0.154	0.013	0.010
1	80000000	56	0.070	0.075	0.094	0.096	0.024	0.021
1	81000000	77	0.106	0.103	0.174	0.172	0.068	0.069
1	82000000	77	0.125	0.150	0.161	0.184	0.036	0.034
1	83000000	82	0.066	0.061	0.127	0.145	0.062	0.084
1	84000000	74	0.128	0.152	0.216	0.206	0.088	0.054
1	85000000	76	0.131	0.141	0.172	0.205	0.042	0.064
1	86000000	83	0.107	0.142	0.196	0.204	0.089	0.063
1	87000000	75	0.088	0.091	0.168	0.149	0.081	0.059
1	88000000	77	0.148	0.154	0.235	0.233	0.087	0.078
1	89000000	92	0.150	0.143	0.168	0.153	0.018	0.011
1	90000000	78	0.152	0.134	0.155	0.157	0.003	0.023
1	91000000	60	0.094	0.110	0.163	0.163	0.069	0.053
1	92000000	75	0.155	0.158	0.111	0.123	0.044	0.035
1	93000000	59	0.119	0.165	0.137	0.140	0.019	0.025
1	94000000	76	0.125	0.150	0.118	0.135	0.008	0.015
1	95000000	72	0.116	0.144	0.143	0.156	0.027	0.012
1	96000000	73	0.095	0.101	0.074	0.092	0.021	0.009
1	97000000	76	0.121	0.124	0.126	0.129	0.005	0.004
1	98000000	51	0.086	0.110	0.092	0.104	0.006	0.006
1	99000000	32	0.141	0.241	0.171	0.175	0.031	0.067

† Australian Working Kelpie (AWK)

‡ Australian Kelpie (AK)

£ minor allele frequency (MAF)

1	100000000	52	0.173	0.138	0.101	0.148	0.072	0.010
1	101000000	48	0.254	0.217	0.149	0.286	0.105	0.069
1	102000000	56	0.137	0.107	0.173	0.174	0.035	0.067
1	103000000	44	0.150	0.160	0.134	0.144	0.016	0.016
1	104000000	22	0.099	0.195	0.155	0.128	0.056	0.067
1	105000000	53	0.108	0.113	0.113	0.115	0.004	0.002
1	106000000	68	0.159	0.147	0.153	0.135	0.006	0.012
1	107000000	59	0.083	0.095	0.115	0.110	0.032	0.015
1	108000000	60	0.184	0.196	0.119	0.149	0.065	0.047
1	109000000	48	0.121	0.135	0.106	0.152	0.015	0.018
1	110000000	53	0.162	0.168	0.154	0.161	0.009	0.007
1	111000000	51	0.152	0.148	0.100	0.102	0.052	0.047
1	112000000	60	0.119	0.122	0.087	0.126	0.032	0.005
1	113000000	71	0.144	0.160	0.160	0.172	0.015	0.012
1	114000000	60	0.058	0.030	0.048	0.062	0.010	0.032
1	115000000	57	0.127	0.131	0.158	0.170	0.031	0.040
1	116000000	54	0.117	0.094	0.105	0.104	0.013	0.010
1	117000000	72	0.183	0.163	0.201	0.186	0.018	0.023
1	118000000	85	0.138	0.128	0.159	0.160	0.021	0.032
1	119000000	72	0.164	0.161	0.157	0.165	0.006	0.004
1	120000000	71	0.176	0.211	0.185	0.184	0.008	0.027
1	121000000	62	0.151	0.167	0.191	0.185	0.040	0.019
1	122000000	46	0.132	0.143	0.156	0.155	0.023	0.011
1	123000000			0.409		0.413	0.000	0.003
2	0	38	0.125	0.069	0.085	0.095	0.040	0.026
2	1000000	68	0.159	0.175	0.196	0.199	0.037	0.024
2	2000000	62	0.222	0.238	0.135	0.164	0.087	0.074
2	3000000	69	0.117	0.123	0.080	0.094	0.037	0.029
2	4000000	7	0.101	0.153	0.148	0.177	0.047	0.024
2	5000000	3	0.102	0.138	0.069	0.104	0.032	0.034
2	6000000	28	0.074	0.110	0.069	0.081	0.005	0.029
2	7000000	66	0.177	0.155	0.179	0.182	0.003	0.027
2	8000000	85	0.203	0.165	0.199	0.211	0.003	0.046
2	9000000	77	0.224	0.200	0.212	0.214	0.012	0.014
2	10000000	70	0.225	0.229	0.171	0.184	0.054	0.046
2	11000000	68	0.141	0.138	0.196	0.192	0.055	0.054
2	12000000	79	0.141	0.117	0.200	0.194	0.059	0.077
2	13000000	40	0.060	0.046	0.094	0.136	0.034	0.090
2	14000000	46	0.120	0.106	0.113	0.137	0.007	0.031
2	15000000	60	0.093	0.097	0.150	0.163	0.057	0.066
2	16000000	65	0.160	0.152	0.147	0.189	0.012	0.038
2	17000000	65	0.122	0.123	0.114	0.137	0.007	0.014
2	18000000	52	0.184	0.130	0.189	0.220	0.005	0.090
2	19000000	71	0.126	0.096	0.152	0.164	0.026	0.068
2	20000000	52	0.133	0.116	0.135	0.161	0.002	0.045
2	21000000	65	0.078	0.064	0.156	0.146	0.078	0.082
2	22000000	86	0.127	0.103	0.111	0.126	0.016	0.023
2	23000000	55	0.102	0.096	0.101	0.142	0.001	0.046
2	24000000	74	0.177	0.180	0.202	0.207	0.025	0.026
2	25000000	69	0.183	0.187	0.156	0.191	0.027	0.004
2	26000000	65	0.145	0.156	0.128	0.152	0.017	0.004
2	27000000	70	0.238	0.211	0.176	0.207	0.062	0.004
2	28000000	63	0.122	0.148	0.093	0.130	0.029	0.019
2	29000000	56	0.124	0.114	0.075	0.105	0.049	0.009
2	30000000	59	0.198	0.193	0.125	0.227	0.074	0.034
2	31000000	72	0.130	0.110	0.123	0.124	0.007	0.014
2	32000000	77	0.148	0.153	0.110	0.128	0.038	0.025
2	33000000	63	0.176	0.167	0.165	0.171	0.011	0.004
2	34000000	56	0.189	0.169	0.111	0.178	0.077	0.009
2	35000000	51	0.195	0.208	0.121	0.168	0.074	0.040
2	36000000	48	0.110	0.094	0.056	0.081	0.054	0.013
2	37000000	58	0.140	0.174	0.084	0.135	0.056	0.038
2	38000000	71	0.167	0.164	0.176	0.207	0.009	0.042
2	39000000	69	0.130	0.162	0.092	0.124	0.038	0.038
2	40000000	70	0.171	0.207	0.129	0.102	0.042	0.104
2	41000000	73	0.161	0.173	0.116	0.112	0.045	0.061
2	42000000	61	0.187	0.190	0.198	0.155	0.012	0.034
2	43000000	47	0.130	0.136	0.131	0.115	0.001	0.021
2	44000000	69	0.137	0.143	0.121	0.116	0.017	0.027
2	45000000	64	0.153	0.151	0.156	0.152	0.003	0.001
2	46000000	58	0.112	0.121	0.121	0.143	0.010	0.022
2	47000000	63	0.145	0.150	0.112	0.108	0.033	0.042
2	48000000	77	0.101	0.085	0.101	0.109	0.000	0.024
2	49000000	65	0.154	0.195	0.208	0.205	0.055	0.010
2	50000000	67	0.173	0.180	0.144	0.172	0.029	0.008
2	51000000	60	0.188	0.166	0.174	0.186	0.014	0.021
2	52000000	68	0.123	0.155	0.120	0.113	0.003	0.043
2	53000000	74	0.097	0.091	0.125	0.164	0.028	0.073
2	54000000	67	0.189	0.171	0.150	0.167	0.039	0.004
2	55000000	71	0.152	0.159	0.107	0.140	0.044	0.019
2	56000000	82	0.210	0.190	0.165	0.172	0.045	0.018
2	57000000	40	0.161	0.134	0.102	0.129	0.059	0.005
2	58000000	68	0.160	0.110	0.110	0.187	0.051	0.077
2	59000000	63	0.100	0.114	0.096	0.148	0.004	0.034
2	60000000	62	0.088	0.094	0.110	0.110	0.022	0.017
2	61000000	71	0.181	0.156	0.164	0.199	0.017	0.043
2	62000000	67	0.192	0.139	0.142	0.167	0.050	0.027
2	63000000	88	0.164	0.139	0.148	0.204	0.015	0.066
2	64000000	71	0.132	0.137	0.147	0.156	0.015	0.019
2	65000000	76	0.127	0.129	0.124	0.154	0.003	0.025
2	66000000	66	0.210	0.195	0.114	0.136	0.096	0.059
2	67000000	74	0.170	0.177	0.135	0.171	0.036	0.006
2	68000000	68	0.132	0.132	0.109	0.129	0.023	0.003
2	69000000	65	0.152	0.138	0.130	0.162	0.022	0.024
2	70000000	66	0.180	0.138	0.152	0.207	0.028	0.070
2	71000000	52	0.113	0.117	0.101	0.117	0.013	0.000
2	72000000	68	0.136	0.110	0.128	0.147	0.008	0.037
2	73000000	61	0.100	0.092	0.139	0.154	0.040	0.062
2	74000000	81	0.145	0.129	0.158	0.152	0.013	0.024
2	75000000	77	0.161	0.128	0.218	0.218	0.057	0.090
2	76000000	64	0.150	0.175	0.196	0.197	0.046	0.021
2	77000000	80	0.144	0.134	0.113	0.124	0.032	0.010
2	78000000	88	0.132	0.140	0.190	0.171	0.058	0.032
2	79000000	70	0.142	0.139	0.208	0.214	0.066	0.075
2	80000000	73	0.184	0.179	0.155	0.160	0.029	0.019

2	81000000	62	0.152	0.190	0.169	0.183	0.017	0.006
2	82000000	78	0.139	0.169	0.116	0.156	0.023	0.012
2	83000000	59	0.139	0.174	0.141	0.196	0.002	0.022
2	84000000	54	0.187	0.174	0.119	0.148	0.068	0.026
2	85000000	23	0.055	0.054	0.033	0.043	0.023	0.011
3	0	59	0.125	0.081	0.081	0.066	0.044	0.015
3	1000000	73	0.126	0.196	0.065	0.168	0.061	0.028
3	2000000	77	0.075	0.079	0.049	0.072	0.025	0.007
3	3000000	62	0.093	0.117	0.099	0.093	0.006	0.024
3	4000000	68	0.102	0.091	0.152	0.133	0.049	0.042
3	5000000	58	0.059	0.076	0.161	0.155	0.102	0.079
3	6000000	76	0.116	0.152	0.191	0.180	0.075	0.028
3	7000000	82	0.157	0.149	0.135	0.182	0.021	0.034
3	8000000	75	0.101	0.175	0.026	0.072	0.075	0.103
3	9000000	62	0.168	0.174	0.075	0.154	0.093	0.021
3	10000000	66	0.150	0.182	0.138	0.171	0.012	0.011
3	11000000	70	0.184	0.185	0.146	0.158	0.038	0.027
3	12000000	71	0.221	0.185	0.174	0.223	0.047	0.038
3	13000000	61	0.161	0.215	0.150	0.213	0.012	0.002
3	14000000	76	0.164	0.188	0.117	0.149	0.046	0.039
3	15000000	81	0.069	0.094	0.089	0.080	0.020	0.014
3	16000000	74	0.126	0.129	0.126	0.171	0.000	0.042
3	17000000	76	0.128	0.141	0.164	0.176	0.037	0.035
3	18000000	50	0.091	0.118	0.142	0.149	0.052	0.030
3	19000000	71	0.129	0.116	0.111	0.156	0.018	0.041
3	20000000	61	0.177	0.195	0.155	0.174	0.022	0.021
3	21000000	59	0.160	0.167	0.143	0.190	0.017	0.024
3	22000000	45	0.078	0.105	0.108	0.137	0.030	0.032
3	23000000	63	0.112	0.128	0.098	0.113	0.014	0.016
3	24000000	63	0.142	0.141	0.089	0.139	0.052	0.002
3	25000000	61	0.143	0.172	0.065	0.071	0.077	0.101
3	26000000	57	0.176	0.187	0.012	0.027	0.164	0.160
3	27000000	66	0.194	0.187	0.012	0.031	0.181	0.156
3	28000000	76	0.118	0.113	0.014	0.019	0.104	0.094
3	29000000	69	0.178	0.151	0.009	0.015	0.169	0.135
3	30000000	77	0.161	0.178	0.008	0.082	0.153	0.096
3	31000000	38	0.146	0.178	0.024	0.101	0.122	0.077
3	32000000	50	0.105	0.123	0.130	0.121	0.026	0.003
3	33000000	56	0.160	0.120	0.140	0.132	0.019	0.012
3	34000000	57	0.129	0.214	0.124	0.118	0.005	0.095
3	35000000	50	0.125	0.135	0.085	0.087	0.040	0.048
3	36000000	47	0.134	0.145	0.069	0.089	0.065	0.056
3	37000000	71	0.133	0.122	0.083	0.105	0.050	0.016
3	38000000	66	0.153	0.150	0.071	0.066	0.082	0.084
3	39000000	65	0.170	0.209	0.085	0.074	0.086	0.135
3	40000000	77	0.123	0.112	0.042	0.050	0.081	0.062
3	41000000	68	0.140	0.160	0.063	0.115	0.076	0.045
3	42000000	71	0.150	0.166	0.188	0.203	0.038	0.037
3	43000000	61	0.120	0.143	0.110	0.122	0.010	0.021
3	44000000	74	0.215	0.204	0.117	0.133	0.098	0.071
3	45000000	63	0.182	0.172	0.109	0.125	0.074	0.047
3	46000000	62	0.111	0.099	0.120	0.118	0.009	0.019
3	47000000	60	0.106	0.119	0.092	0.117	0.014	0.001
3	48000000	70	0.156	0.152	0.157	0.179	0.001	0.028
3	49000000	77	0.142	0.117	0.122	0.169	0.020	0.052
3	50000000	79	0.117	0.093	0.113	0.114	0.004	0.022
3	51000000	72	0.152	0.130	0.113	0.152	0.039	0.022
3	52000000	67	0.133	0.123	0.170	0.164	0.037	0.041
3	53000000	62	0.133	0.144	0.133	0.163	0.000	0.019
3	54000000	73	0.214	0.196	0.062	0.063	0.152	0.133
3	55000000	65	0.129	0.173	0.168	0.194	0.039	0.021
3	56000000	79	0.124	0.104	0.119	0.119	0.005	0.015
3	57000000	78	0.150	0.136	0.168	0.189	0.018	0.053
3	58000000	64	0.131	0.143	0.127	0.131	0.004	0.012
3	59000000	82	0.171	0.157	0.209	0.221	0.038	0.064
3	60000000	78	0.118	0.133	0.123	0.150	0.005	0.017
3	61000000	70	0.118	0.124	0.086	0.113	0.032	0.011
3	62000000	74	0.218	0.220	0.112	0.168	0.106	0.052
3	63000000	73	0.124	0.109	0.125	0.147	0.001	0.038
3	64000000	87	0.115	0.110	0.129	0.173	0.014	0.063
3	65000000	68	0.144	0.176	0.169	0.165	0.025	0.011
3	66000000	78	0.160	0.173	0.184	0.189	0.023	0.016
3	67000000	76	0.246	0.248	0.211	0.196	0.036	0.052
3	68000000	80	0.218	0.212	0.180	0.178	0.038	0.034
3	69000000	80	0.186	0.184	0.143	0.140	0.043	0.044
3	70000000	69	0.231	0.206	0.143	0.143	0.087	0.064
3	71000000	68	0.134	0.188	0.165	0.163	0.031	0.025
3	72000000	71	0.099	0.103	0.089	0.116	0.010	0.013
3	73000000	68	0.106	0.127	0.167	0.178	0.061	0.051
3	74000000	73	0.114	0.177	0.145	0.225	0.031	0.049
3	75000000	75	0.131	0.102	0.145	0.146	0.013	0.044
3	76000000	69	0.187	0.160	0.247	0.268	0.059	0.108
3	77000000	77	0.184	0.164	0.191	0.218	0.008	0.054
3	78000000	76	0.182	0.183	0.220	0.234	0.038	0.051
3	79000000	79	0.175	0.187	0.143	0.180	0.032	0.007
3	80000000	79	0.175	0.181	0.149	0.180	0.026	0.001
3	81000000	64	0.207	0.204	0.121	0.156	0.086	0.048
3	82000000	75	0.149	0.167	0.104	0.124	0.046	0.043
3	83000000	73	0.217	0.226	0.153	0.201	0.064	0.025
3	84000000	79	0.160	0.149	0.148	0.182	0.013	0.033
3	85000000	75	0.194	0.197	0.182	0.205	0.012	0.008
3	86000000	77	0.209	0.207	0.231	0.243	0.022	0.036
3	87000000	73	0.213	0.219	0.189	0.204	0.024	0.015
3	88000000	90	0.180	0.177	0.182	0.200	0.003	0.023
3	89000000	79	0.204	0.191	0.168	0.155	0.036	0.036
3	90000000	85	0.162	0.164	0.181	0.190	0.019	0.025
3	91000000	59	0.149	0.165	0.090	0.104	0.059	0.061
3	92000000	2	0.021	0.011	0.125	0.119	0.104	0.107
4	0	65	0.169	0.146	0.166	0.164	0.002	0.018
4	1000000	66	0.167	0.127	0.089	0.138	0.078	0.011
4	2000000	73	0.158	0.133	0.118	0.126	0.040	0.007
4	3000000	64	0.154	0.116	0.088	0.090	0.066	0.026
4	4000000	54	0.159	0.203	0.155	0.148	0.004	0.054
4	5000000	76	0.202	0.193	0.165	0.133	0.037	0.060
4	6000000	89	0.111	0.071	0.083	0.099	0.028	0.028

4	7000000	84	0.101	0.098	0.126	0.141	0.025	0.044
4	8000000	72	0.143	0.131	0.133	0.118	0.010	0.013
4	9000000	80	0.159	0.147	0.141	0.149	0.018	0.002
4	10000000	65	0.220	0.219	0.173	0.174	0.047	0.045
4	11000000	70	0.193	0.163	0.131	0.132	0.063	0.031
4	12000000	77	0.184	0.190	0.149	0.149	0.035	0.041
4	13000000	64	0.104	0.116	0.088	0.095	0.016	0.021
4	14000000	64	0.130	0.104	0.138	0.141	0.007	0.037
4	15000000	57	0.117	0.126	0.112	0.095	0.005	0.031
4	16000000	65	0.185	0.179	0.160	0.149	0.026	0.030
4	17000000	65	0.159	0.158	0.155	0.171	0.004	0.012
4	18000000	71	0.148	0.140	0.126	0.114	0.022	0.026
4	19000000	51	0.152	0.123	0.125	0.121	0.027	0.002
4	20000000	63	0.184	0.132	0.122	0.153	0.062	0.021
4	21000000	72	0.200	0.180	0.152	0.152	0.048	0.028
4	22000000	69	0.174	0.136	0.182	0.191	0.008	0.055
4	23000000	60	0.158	0.137	0.129	0.144	0.029	0.007
4	24000000	65	0.188	0.163	0.161	0.137	0.027	0.026
4	25000000	66	0.124	0.079	0.135	0.135	0.012	0.056
4	26000000	69	0.165	0.154	0.150	0.189	0.015	0.035
4	27000000	78	0.119	0.115	0.134	0.122	0.016	0.007
4	28000000	77	0.100	0.111	0.123	0.103	0.023	0.008
4	29000000	81	0.123	0.131	0.153	0.177	0.030	0.046
4	30000000	81	0.145	0.156	0.209	0.201	0.064	0.045
4	31000000	73	0.178	0.144	0.195	0.200	0.017	0.056
4	32000000	68	0.149	0.096	0.197	0.193	0.047	0.096
4	33000000	65	0.162	0.144	0.182	0.179	0.021	0.035
4	34000000	63	0.112	0.071	0.171	0.161	0.059	0.090
4	35000000	75	0.159	0.096	0.171	0.166	0.012	0.070
4	36000000	68	0.186	0.173	0.162	0.182	0.025	0.009
4	37000000	66	0.122	0.097	0.177	0.166	0.055	0.069
4	38000000	79	0.159	0.134	0.172	0.173	0.013	0.040
4	39000000	54	0.074	0.125	0.137	0.180	0.063	0.055
4	40000000	59	0.054	0.071	0.126	0.122	0.073	0.051
4	41000000	68	0.045	0.074	0.098	0.144	0.053	0.069
4	42000000	71	0.135	0.132	0.147	0.185	0.011	0.053
4	43000000	81	0.168	0.181	0.166	0.200	0.002	0.020
4	44000000	73	0.151	0.155	0.177	0.179	0.026	0.024
4	45000000	62	0.166	0.205	0.228	0.204	0.062	0.001
4	46000000	61	0.126	0.116	0.133	0.137	0.007	0.022
4	47000000	71	0.168	0.192	0.179	0.195	0.011	0.003
4	48000000	78	0.163	0.139	0.190	0.215	0.027	0.076
4	49000000	69	0.111	0.116	0.122	0.130	0.011	0.014
4	50000000	71	0.102	0.105	0.140	0.163	0.039	0.058
4	51000000	75	0.089	0.100	0.150	0.144	0.060	0.044
4	52000000	74	0.137	0.143	0.174	0.162	0.037	0.018
4	53000000	67	0.114	0.117	0.146	0.144	0.032	0.027
4	54000000	74	0.158	0.162	0.144	0.177	0.013	0.016
4	55000000	70	0.128	0.114	0.154	0.175	0.026	0.061
4	56000000	69	0.129	0.128	0.161	0.195	0.033	0.067
4	57000000	80	0.190	0.186	0.172	0.198	0.018	0.012
4	58000000	80	0.095	0.100	0.148	0.155	0.053	0.055
4	59000000	81	0.147	0.156	0.126	0.140	0.021	0.016
4	60000000	64	0.101	0.116	0.127	0.144	0.026	0.028
4	61000000	61	0.107	0.092	0.166	0.159	0.059	0.067
4	62000000	69	0.150	0.153	0.176	0.164	0.026	0.011
4	63000000	73	0.157	0.165	0.151	0.133	0.006	0.032
4	64000000	69	0.148	0.135	0.120	0.144	0.028	0.009
4	65000000	59	0.144	0.160	0.141	0.137	0.004	0.023
4	66000000	66	0.143	0.149	0.147	0.178	0.004	0.030
4	67000000	67	0.168	0.176	0.099	0.116	0.068	0.059
4	68000000	75	0.123	0.125	0.161	0.180	0.038	0.055
4	69000000	81	0.168	0.148	0.179	0.194	0.012	0.047
4	70000000	83	0.144	0.151	0.146	0.171	0.002	0.020
4	71000000	83	0.162	0.147	0.167	0.182	0.005	0.035
4	72000000	65	0.194	0.207	0.211	0.242	0.017	0.035
4	73000000	82	0.170	0.175	0.189	0.203	0.018	0.028
4	74000000	70	0.156	0.170	0.119	0.105	0.036	0.064
4	75000000	65	0.171	0.185	0.133	0.204	0.038	0.019
4	76000000	77	0.145	0.154	0.108	0.125	0.037	0.029
4	77000000	66	0.141	0.159	0.117	0.135	0.025	0.024
4	78000000	84	0.185	0.156	0.125	0.133	0.060	0.023
4	79000000	71	0.170	0.169	0.077	0.126	0.093	0.043
4	80000000	76	0.184	0.191	0.101	0.138	0.083	0.053
4	81000000	78	0.207	0.219	0.161	0.210	0.046	0.009
4	82000000	74	0.211	0.214	0.127	0.198	0.084	0.016
4	83000000	80	0.200	0.196	0.087	0.199	0.112	0.003
4	84000000	79	0.171	0.155	0.091	0.150	0.080	0.005
4	85000000	72	0.174	0.167	0.129	0.157	0.045	0.010
4	86000000	72	0.180	0.192	0.134	0.180	0.046	0.012
4	87000000	79	0.192	0.169	0.191	0.189	0.001	0.020
4	88000000	20	0.173	0.161	0.143	0.209	0.030	0.048
4	89000000			0.318	0.042	0.167	0.042	0.152
5	0	51	0.110	0.113	0.092	0.108	0.018	0.005
5	1000000	56	0.060	0.046	0.072	0.096	0.012	0.050
5	2000000	89	0.075	0.078	0.065	0.077	0.010	0.002
5	3000000	75	0.196	0.162	0.137	0.139	0.058	0.023
5	4000000	80	0.096	0.083	0.153	0.093	0.057	0.009
5	5000000	76	0.168	0.174	0.204	0.205	0.036	0.031
5	6000000	69	0.137	0.133	0.149	0.156	0.012	0.023
5	7000000	77	0.101	0.107	0.086	0.077	0.015	0.030
5	8000000	69	0.162	0.141	0.167	0.173	0.005	0.032
5	9000000	66	0.142	0.134	0.143	0.161	0.000	0.027
5	10000000	59	0.175	0.188	0.199	0.191	0.023	0.003
5	11000000	57	0.200	0.154	0.165	0.169	0.036	0.015
5	12000000	68	0.139	0.138	0.159	0.141	0.020	0.003
5	13000000	74	0.189	0.187	0.163	0.172	0.026	0.015
5	14000000	68	0.176	0.192	0.187	0.175	0.011	0.017
5	15000000	66	0.188	0.188	0.191	0.192	0.003	0.003
5	16000000	67	0.189	0.202	0.232	0.244	0.043	0.042
5	17000000	81	0.132	0.145	0.174	0.182	0.042	0.038
5	18000000	56	0.208	0.200	0.164	0.194	0.043	0.006
5	19000000	74	0.145	0.154	0.163	0.161	0.018	0.007
5	20000000	57	0.155	0.190	0.205	0.201	0.050	0.012
5	21000000	66	0.136	0.138	0.108	0.136	0.028	0.002

5	22000000	61	0.146	0.137	0.108	0.139	0.039	0.003
5	23000000	57	0.204	0.233	0.213	0.229	0.008	0.004
5	24000000	67	0.183	0.205	0.173	0.196	0.010	0.009
5	25000000	67	0.180	0.188	0.163	0.192	0.016	0.004
5	26000000	68	0.176	0.175	0.210	0.201	0.034	0.026
5	27000000	80	0.174	0.145	0.167	0.192	0.007	0.047
5	28000000	86	0.179	0.171	0.169	0.179	0.010	0.008
5	29000000	68	0.151	0.184	0.180	0.199	0.029	0.015
5	30000000	64	0.064	0.107	0.129	0.129	0.065	0.022
5	31000000	68	0.106	0.137	0.168	0.178	0.062	0.042
5	32000000	61	0.063	0.067	0.138	0.160	0.075	0.093
5	33000000	76	0.136	0.136	0.177	0.187	0.041	0.051
5	34000000	60	0.102	0.116	0.134	0.147	0.031	0.030
5	35000000	74	0.149	0.198	0.179	0.211	0.030	0.013
5	36000000	70	0.132	0.144	0.144	0.171	0.012	0.027
5	37000000	79	0.119	0.153	0.103	0.151	0.016	0.002
5	38000000	72	0.103	0.127	0.119	0.131	0.015	0.004
5	39000000	56	0.158	0.144	0.078	0.143	0.081	0.001
5	40000000	59	0.102	0.088	0.063	0.083	0.039	0.004
5	41000000	79	0.173	0.196	0.122	0.165	0.051	0.030
5	42000000	43	0.124	0.161	0.149	0.173	0.025	0.011
5	43000000	68	0.144	0.147	0.160	0.161	0.016	0.014
5	44000000	72	0.181	0.188	0.179	0.190	0.002	0.002
5	45000000	76	0.153	0.144	0.174	0.168	0.020	0.024
5	46000000	81	0.128	0.103	0.148	0.158	0.020	0.055
5	47000000	78	0.145	0.141	0.134	0.102	0.011	0.039
5	48000000	76	0.171	0.152	0.139	0.152	0.032	0.000
5	49000000	62	0.202	0.209	0.148	0.176	0.055	0.033
5	50000000	68	0.142	0.144	0.158	0.181	0.016	0.038
5	51000000	67	0.172	0.176	0.199	0.221	0.026	0.045
5	52000000	63	0.184	0.179	0.139	0.169	0.045	0.010
5	53000000	62	0.146	0.153	0.147	0.166	0.001	0.013
5	54000000	62	0.138	0.144	0.147	0.155	0.009	0.010
5	55000000	58	0.172	0.187	0.116	0.141	0.056	0.046
5	56000000	53	0.056	0.068	0.130	0.115	0.074	0.047
5	57000000	77	0.164	0.183	0.151	0.144	0.014	0.039
5	58000000	66	0.139	0.156	0.182	0.172	0.044	0.017
5	59000000	87	0.152	0.154	0.147	0.135	0.005	0.019
5	60000000	68	0.106	0.125	0.084	0.106	0.022	0.019
5	61000000	67	0.123	0.128	0.088	0.074	0.035	0.054
5	62000000	62	0.145	0.160	0.147	0.153	0.003	0.006
5	63000000	73	0.134	0.144	0.160	0.160	0.026	0.016
5	64000000	67	0.140	0.172	0.136	0.153	0.004	0.019
5	65000000	68	0.157	0.193	0.204	0.211	0.046	0.018
5	66000000	59	0.131	0.149	0.163	0.201	0.032	0.052
5	67000000	74	0.170	0.159	0.156	0.163	0.013	0.004
5	68000000	73	0.200	0.206	0.192	0.221	0.008	0.015
5	69000000	76	0.219	0.221	0.196	0.205	0.023	0.016
5	70000000	77	0.149	0.155	0.136	0.192	0.013	0.037
5	71000000	74	0.155	0.183	0.144	0.184	0.011	0.000
5	72000000	85	0.160	0.175	0.175	0.213	0.015	0.038
5	73000000	71	0.168	0.186	0.140	0.166	0.029	0.019
5	74000000	68	0.126	0.107	0.157	0.183	0.030	0.076
5	75000000	74	0.098	0.107	0.161	0.127	0.063	0.020
5	76000000	53	0.117	0.147	0.130	0.130	0.013	0.017
5	77000000	61	0.067	0.059	0.123	0.117	0.056	0.058
5	78000000	62	0.203	0.150	0.165	0.197	0.038	0.047
5	79000000	62	0.159	0.128	0.141	0.124	0.019	0.004
5	80000000	48	0.143	0.130	0.122	0.132	0.021	0.002
5	81000000	41	0.140	0.119	0.113	0.194	0.027	0.075
5	82000000	65	0.112	0.089	0.091	0.123	0.021	0.033
5	83000000	83	0.165	0.116	0.145	0.194	0.021	0.078
5	84000000	67	0.115	0.096	0.152	0.185	0.037	0.089
5	85000000	73	0.142	0.112	0.137	0.161	0.006	0.049
5	86000000	77	0.149	0.144	0.203	0.221	0.054	0.077
5	87000000	68	0.142	0.138	0.182	0.187	0.039	0.049
5	88000000	63	0.186	0.153	0.205	0.211	0.019	0.058
5	89000000	2	0.063	0.136	0.167	0.175	0.104	0.039
6	0	34	0.091	0.099	0.110	0.126	0.019	0.027
6	1000000	59	0.163	0.162	0.221	0.240	0.058	0.078
6	2000000	61	0.102	0.133	0.151	0.147	0.049	0.014
6	3000000	80	0.145	0.127	0.130	0.151	0.016	0.024
6	4000000	64	0.190	0.174	0.126	0.157	0.064	0.017
6	5000000	78	0.069	0.096	0.100	0.128	0.031	0.032
6	6000000	74	0.083	0.090	0.217	0.214	0.134	0.124
6	7000000	78	0.162	0.177	0.160	0.211	0.002	0.034
6	8000000	80	0.209	0.209	0.146	0.184	0.063	0.025
6	9000000	77	0.153	0.154	0.153	0.170	0.000	0.016
6	10000000	71	0.208	0.221	0.169	0.201	0.040	0.021
6	11000000	56	0.180	0.186	0.228	0.230	0.048	0.044
6	12000000	70	0.171	0.141	0.177	0.167	0.006	0.026
6	13000000	61	0.115	0.115	0.176	0.201	0.061	0.086
6	14000000	72	0.061	0.039	0.126	0.118	0.065	0.079
6	15000000	90	0.114	0.063	0.142	0.160	0.027	0.098
6	16000000	78	0.124	0.097	0.190	0.214	0.065	0.117
6	17000000	63	0.057	0.051	0.144	0.185	0.087	0.134
6	18000000	59	0.117	0.127	0.123	0.142	0.006	0.015
6	19000000	67	0.132	0.133	0.156	0.204	0.024	0.072
6	20000000	70	0.109	0.142	0.149	0.179	0.040	0.037
6	21000000	69	0.110	0.126	0.139	0.153	0.029	0.027
6	22000000	75	0.039	0.044	0.090	0.105	0.051	0.061
6	23000000	50	0.043	0.055	0.102	0.079	0.059	0.025
6	24000000	45	0.081	0.035	0.130	0.047	0.049	0.012
6	25000000	55	0.070	0.076	0.093	0.123	0.023	0.047
6	26000000	68	0.166	0.166	0.213	0.216	0.047	0.050
6	27000000	67	0.144	0.121	0.146	0.162	0.001	0.040
6	28000000	67	0.144	0.110	0.132	0.130	0.013	0.021
6	29000000	55	0.075	0.090	0.104	0.109	0.029	0.019
6	30000000	70	0.167	0.172	0.110	0.161	0.057	0.011
6	31000000	93	0.151	0.147	0.137	0.148	0.014	0.001
6	32000000	74	0.126	0.071	0.129	0.122	0.002	0.051
6	33000000	79	0.116	0.043	0.176	0.170	0.060	0.127
6	34000000	72	0.068	0.057	0.064	0.083	0.004	0.025
6	35000000	71	0.138	0.137	0.172	0.172	0.033	0.035
6	36000000	65	0.100	0.083	0.055	0.078	0.045	0.005

6	37000000	67	0.105	0.087	0.059	0.090	0.046	0.003
6	38000000	61	0.079	0.108	0.056	0.072	0.023	0.036
6	39000000	66	0.052	0.071	0.083	0.100	0.032	0.029
6	40000000	44	0.027	0.014	0.064	0.066	0.036	0.052
6	41000000	72	0.076	0.107	0.054	0.134	0.023	0.028
6	42000000	82	0.082	0.124	0.073	0.135	0.008	0.011
6	43000000	73	0.167	0.157	0.128	0.134	0.039	0.024
6	44000000	79	0.170	0.166	0.148	0.142	0.021	0.024
6	45000000	73	0.202	0.191	0.142	0.168	0.060	0.023
6	46000000	79	0.163	0.134	0.201	0.243	0.038	0.109
6	47000000	76	0.111	0.083	0.136	0.168	0.026	0.085
6	48000000	84	0.157	0.125	0.131	0.161	0.026	0.036
6	49000000	71	0.197	0.206	0.155	0.164	0.042	0.041
6	50000000	70	0.134	0.139	0.137	0.159	0.003	0.020
6	51000000	74	0.168	0.169	0.152	0.160	0.016	0.008
6	52000000	65	0.172	0.174	0.129	0.175	0.043	0.002
6	53000000	73	0.121	0.121	0.124	0.141	0.003	0.020
6	54000000	67	0.225	0.204	0.200	0.202	0.024	0.001
6	55000000	72	0.183	0.199	0.173	0.168	0.009	0.031
6	56000000	70	0.164	0.211	0.149	0.133	0.015	0.077
6	57000000	71	0.171	0.185	0.155	0.126	0.016	0.059
6	58000000	72	0.129	0.143	0.133	0.145	0.004	0.002
6	59000000	70	0.171	0.175	0.121	0.159	0.050	0.016
6	60000000	70	0.197	0.187	0.169	0.165	0.028	0.022
6	61000000	51	0.175	0.164	0.054	0.128	0.120	0.036
6	62000000	65	0.152	0.153	0.126	0.181	0.026	0.028
6	63000000	74	0.158	0.147	0.144	0.153	0.014	0.006
6	64000000	70	0.190	0.203	0.167	0.218	0.023	0.015
6	65000000	84	0.156	0.168	0.161	0.164	0.005	0.005
6	66000000	73	0.150	0.174	0.166	0.170	0.017	0.004
6	67000000	79	0.142	0.150	0.155	0.179	0.014	0.029
6	68000000	70	0.123	0.138	0.145	0.185	0.022	0.047
6	69000000	84	0.110	0.104	0.179	0.186	0.069	0.082
6	70000000	88	0.129	0.114	0.150	0.170	0.021	0.056
6	71000000	74	0.087	0.081	0.111	0.136	0.024	0.055
6	72000000	61	0.166	0.160	0.171	0.178	0.005	0.018
6	73000000	65	0.127	0.127	0.172	0.169	0.046	0.042
6	74000000	84	0.147	0.146	0.164	0.204	0.017	0.058
6	75000000	67	0.147	0.141	0.183	0.214	0.037	0.073
6	76000000	77	0.125	0.141	0.166	0.157	0.041	0.016
6	77000000	52	0.101	0.122	0.205	0.190	0.104	0.068
7	0	61	0.183	0.187	0.115	0.133	0.068	0.054
7	1000000	50	0.174	0.141	0.214	0.179	0.041	0.039
7	2000000	71	0.144	0.134	0.130	0.114	0.014	0.020
7	3000000	77	0.251	0.219	0.226	0.239	0.025	0.020
7	4000000	79	0.138	0.125	0.093	0.133	0.045	0.008
7	5000000	74	0.168	0.182	0.069	0.121	0.099	0.061
7	6000000	74	0.165	0.157	0.133	0.193	0.032	0.036
7	7000000	81	0.152	0.142	0.131	0.168	0.021	0.026
7	8000000	77	0.184	0.167	0.164	0.188	0.020	0.021
7	9000000	62	0.180	0.188	0.135	0.179	0.046	0.008
7	10000000	70	0.144	0.128	0.104	0.158	0.040	0.031
7	11000000	80	0.211	0.194	0.154	0.183	0.057	0.010
7	12000000	71	0.165	0.161	0.118	0.154	0.047	0.008
7	13000000	61	0.148	0.149	0.098	0.144	0.050	0.006
7	14000000	79	0.160	0.158	0.103	0.127	0.056	0.031
7	15000000	69	0.178	0.190	0.110	0.136	0.068	0.054
7	16000000	74	0.167	0.145	0.102	0.131	0.065	0.014
7	17000000	74	0.178	0.171	0.127	0.145	0.051	0.026
7	18000000	65	0.170	0.184	0.129	0.151	0.041	0.033
7	19000000	67	0.166	0.156	0.135	0.166	0.031	0.010
7	20000000	62	0.173	0.168	0.161	0.179	0.012	0.010
7	21000000	67	0.117	0.120	0.105	0.109	0.012	0.011
7	22000000	77	0.213	0.222	0.172	0.205	0.041	0.017
7	23000000	64	0.202	0.186	0.141	0.162	0.061	0.025
7	24000000	53	0.199	0.185	0.209	0.225	0.010	0.040
7	25000000	60	0.106	0.101	0.133	0.146	0.027	0.045
7	26000000	67	0.155	0.128	0.148	0.181	0.007	0.053
7	27000000	60	0.136	0.152	0.191	0.219	0.055	0.067
7	28000000	67	0.130	0.135	0.136	0.147	0.005	0.012
7	29000000	75	0.165	0.157	0.169	0.170	0.004	0.013
7	30000000	78	0.155	0.173	0.146	0.140	0.009	0.033
7	31000000	59	0.161	0.167	0.169	0.176	0.009	0.009
7	32000000	74	0.209	0.219	0.103	0.120	0.106	0.099
7	33000000	72	0.232	0.213	0.162	0.155	0.070	0.058
7	34000000	69	0.087	0.084	0.129	0.118	0.042	0.034
7	35000000	76	0.165	0.163	0.189	0.173	0.024	0.010
7	36000000	68	0.189	0.191	0.113	0.131	0.076	0.061
7	37000000	46	0.134	0.136	0.160	0.175	0.026	0.039
7	38000000	66	0.154	0.130	0.140	0.175	0.014	0.045
7	39000000	69	0.194	0.201	0.153	0.163	0.041	0.038
7	40000000	63	0.149	0.167	0.146	0.122	0.004	0.045
7	41000000	69	0.089	0.079	0.139	0.114	0.050	0.035
7	42000000	61	0.090	0.082	0.051	0.045	0.039	0.037
7	43000000	74	0.243	0.200	0.201	0.168	0.042	0.032
7	44000000	72	0.143	0.151	0.123	0.114	0.019	0.038
7	45000000	76	0.182	0.161	0.155	0.161	0.027	0.000
7	46000000	68	0.173	0.154	0.169	0.172	0.004	0.017
7	47000000	76	0.183	0.161	0.158	0.148	0.025	0.013
7	48000000	74	0.157	0.147	0.144	0.113	0.013	0.034
7	49000000	61	0.212	0.210	0.185	0.186	0.028	0.024
7	50000000	74	0.114	0.140	0.117	0.094	0.004	0.047
7	51000000	82	0.093	0.091	0.125	0.128	0.032	0.037
7	52000000	69	0.121	0.121	0.121	0.162	0.000	0.041
7	53000000	52	0.087	0.116	0.123	0.147	0.036	0.031
7	54000000	68	0.074	0.142	0.109	0.157	0.035	0.014
7	55000000	73	0.140	0.148	0.200	0.179	0.060	0.030
7	56000000	63	0.129	0.145	0.113	0.086	0.015	0.059
7	57000000	86	0.157	0.140	0.151	0.146	0.007	0.006
7	58000000	69	0.154	0.170	0.190	0.206	0.036	0.035
7	59000000	65	0.150	0.146	0.172	0.218	0.023	0.072
7	60000000	75	0.130	0.120	0.092	0.146	0.038	0.027
7	61000000	78	0.167	0.185	0.085	0.159	0.082	0.026
7	62000000	75	0.155	0.181	0.093	0.138	0.061	0.043
7	63000000	70	0.226	0.215	0.157	0.158	0.069	0.057

7	64000000	71	0.171	0.181	0.162	0.160	0.009	0.021
7	65000000	73	0.164	0.175	0.167	0.188	0.003	0.013
7	66000000	66	0.179	0.173	0.199	0.176	0.020	0.003
7	67000000	71	0.130	0.120	0.124	0.130	0.006	0.011
7	68000000	72	0.106	0.110	0.092	0.102	0.013	0.008
7	69000000	77	0.165	0.149	0.138	0.139	0.027	0.010
7	70000000	72	0.187	0.141	0.177	0.178	0.010	0.037
7	71000000	68	0.158	0.139	0.197	0.218	0.039	0.080
7	72000000	64	0.152	0.176	0.199	0.212	0.047	0.036
7	73000000	76	0.150	0.160	0.230	0.223	0.080	0.064
7	74000000	77	0.174	0.198	0.136	0.153	0.038	0.045
7	75000000	71	0.181	0.178	0.172	0.189	0.008	0.011
7	76000000	82	0.146	0.130	0.191	0.233	0.045	0.103
7	77000000	73	0.163	0.171	0.173	0.207	0.011	0.037
7	78000000	74	0.174	0.166	0.177	0.203	0.004	0.037
7	79000000	68	0.173	0.200	0.204	0.195	0.031	0.005
7	80000000	65	0.146	0.155	0.152	0.169	0.006	0.013
8	0	47	0.128	0.125	0.106	0.115	0.022	0.010
8	1000000	70	0.173	0.158	0.154	0.163	0.019	0.006
8	2000000	72	0.179	0.174	0.139	0.163	0.040	0.011
8	3000000	64	0.136	0.143	0.152	0.185	0.016	0.042
8	4000000	68	0.182	0.209	0.195	0.192	0.014	0.016
8	5000000	68	0.195	0.198	0.203	0.200	0.008	0.002
8	6000000	69	0.183	0.188	0.165	0.156	0.019	0.031
8	7000000	44	0.176	0.234	0.180	0.176	0.004	0.058
8	8000000	67	0.163	0.156	0.144	0.161	0.019	0.005
8	9000000	59	0.182	0.172	0.190	0.203	0.008	0.032
8	10000000	61	0.141	0.123	0.119	0.145	0.022	0.022
8	11000000	69	0.199	0.171	0.209	0.219	0.010	0.048
8	12000000	70	0.184	0.184	0.174	0.169	0.010	0.014
8	13000000	54	0.163	0.129	0.175	0.171	0.013	0.042
8	14000000	61	0.172	0.161	0.209	0.228	0.037	0.067
8	15000000	64	0.164	0.174	0.196	0.219	0.032	0.045
8	16000000	61	0.172	0.154	0.176	0.166	0.004	0.012
8	17000000	63	0.174	0.177	0.241	0.256	0.067	0.079
8	18000000	64	0.144	0.139	0.159	0.154	0.015	0.015
8	19000000	66	0.176	0.162	0.185	0.188	0.009	0.025
8	20000000	66	0.171	0.163	0.167	0.162	0.004	0.000
8	21000000	57	0.126	0.133	0.138	0.135	0.012	0.002
8	22000000	56	0.211	0.214	0.243	0.234	0.032	0.020
8	23000000	63	0.246	0.256	0.190	0.207	0.056	0.049
8	24000000	64	0.152	0.139	0.175	0.190	0.023	0.051
8	25000000	71	0.191	0.185	0.183	0.187	0.008	0.002
8	26000000	67	0.111	0.099	0.152	0.173	0.041	0.074
8	27000000	58	0.149	0.172	0.133	0.161	0.017	0.011
8	28000000	52	0.150	0.157	0.178	0.194	0.028	0.037
8	29000000	65	0.143	0.138	0.155	0.151	0.012	0.013
8	30000000	70	0.178	0.168	0.174	0.176	0.004	0.009
8	31000000	65	0.126	0.133	0.169	0.170	0.043	0.037
8	32000000	59	0.166	0.207	0.160	0.192	0.006	0.014
8	33000000	62	0.136	0.147	0.130	0.132	0.006	0.015
8	34000000	75	0.174	0.173	0.169	0.162	0.005	0.012
8	35000000	57	0.156	0.183	0.196	0.181	0.040	0.001
8	36000000	78	0.152	0.171	0.182	0.182	0.031	0.011
8	37000000	68	0.175	0.186	0.201	0.155	0.026	0.031
8	38000000	65	0.193	0.182	0.259	0.234	0.066	0.052
8	39000000	58	0.094	0.126	0.140	0.145	0.046	0.020
8	40000000	54	0.116	0.164	0.184	0.186	0.068	0.021
8	41000000	64	0.169	0.166	0.184	0.178	0.015	0.012
8	42000000	72	0.230	0.247	0.225	0.227	0.006	0.020
8	43000000	69	0.124	0.112	0.158	0.132	0.034	0.020
8	44000000	55	0.192	0.174	0.151	0.121	0.041	0.053
8	45000000	69	0.155	0.156	0.136	0.129	0.019	0.026
8	46000000	61	0.164	0.159	0.160	0.169	0.004	0.010
8	47000000	63	0.106	0.085	0.131	0.127	0.025	0.042
8	48000000	64	0.103	0.111	0.125	0.133	0.022	0.022
8	49000000	74	0.190	0.190	0.192	0.237	0.002	0.047
8	50000000	59	0.162	0.139	0.130	0.166	0.032	0.027
8	51000000	68	0.149	0.158	0.164	0.161	0.015	0.003
8	52000000	56	0.186	0.169	0.186	0.183	0.001	0.014
8	53000000	56	0.135	0.126	0.103	0.142	0.032	0.016
8	54000000	60	0.215	0.222	0.170	0.192	0.045	0.030
8	55000000	57	0.172	0.173	0.188	0.201	0.016	0.028
8	56000000	59	0.136	0.134	0.161	0.167	0.025	0.033
8	57000000	64	0.196	0.216	0.152	0.200	0.044	0.016
8	58000000	75	0.104	0.105	0.153	0.177	0.049	0.072
8	59000000	54	0.089	0.098	0.143	0.151	0.054	0.054
8	60000000	69	0.110	0.085	0.159	0.169	0.049	0.085
8	61000000	85	0.137	0.116	0.162	0.160	0.025	0.045
8	62000000	64	0.103	0.089	0.143	0.160	0.040	0.071
8	63000000	75	0.100	0.085	0.155	0.158	0.054	0.073
8	64000000	81	0.049	0.049	0.166	0.189	0.118	0.141
8	65000000	75	0.042	0.040	0.165	0.221	0.123	0.182
8	66000000	80	0.035	0.033	0.135	0.184	0.099	0.151
8	67000000	71	0.008	0.004	0.130	0.131	0.122	0.126
8	68000000	72	0.076	0.047	0.169	0.183	0.092	0.137
8	69000000	72	0.053	0.040	0.148	0.173	0.096	0.133
8	70000000	55	0.048	0.036	0.096	0.124	0.047	0.088
8	71000000	62	0.085	0.065	0.138	0.116	0.053	0.051
8	72000000	47	0.118	0.096	0.149	0.167	0.032	0.071
8	73000000	12	0.134	0.066	0.125	0.180	0.008	0.114
8	74000000	2	0.000	0.000	0.136	0.000	0.136	0.000
9	0	50	0.181	0.180	0.197	0.193	0.016	0.013
9	1000000	41	0.145	0.145	0.166	0.177	0.021	0.032
9	2000000	56	0.095	0.119	0.096	0.141	0.001	0.022
9	3000000	56	0.113	0.104	0.072	0.103	0.041	0.001
9	4000000	60	0.157	0.117	0.130	0.152	0.027	0.034
9	5000000	53	0.147	0.114	0.113	0.153	0.034	0.039
9	6000000	62	0.115	0.094	0.107	0.170	0.007	0.076
9	7000000	36	0.147	0.175	0.114	0.149	0.033	0.026
9	8000000	41	0.126	0.127	0.158	0.163	0.032	0.036
9	9000000	63	0.128	0.135	0.117	0.128	0.011	0.007
9	10000000	58	0.077	0.095	0.129	0.176	0.052	0.081
9	11000000	70	0.152	0.157	0.138	0.165	0.014	0.008
9	12000000	71	0.181	0.190	0.158	0.168	0.023	0.022

9	13000000	68	0.115	0.134	0.132	0.188	0.017	0.054
9	14000000	74	0.123	0.114	0.102	0.124	0.020	0.010
9	15000000	79	0.168	0.182	0.176	0.175	0.008	0.007
9	16000000	59	0.121	0.161	0.161	0.182	0.040	0.021
9	17000000	40	0.177	0.158	0.138	0.177	0.039	0.018
9	18000000	44	0.113	0.137	0.158	0.183	0.046	0.046
9	19000000	70	0.105	0.116	0.210	0.177	0.104	0.061
9	20000000	55	0.053	0.089	0.167	0.166	0.114	0.077
9	21000000	56	0.161	0.172	0.244	0.259	0.082	0.087
9	22000000	58	0.126	0.153	0.137	0.159	0.010	0.006
9	23000000	69	0.125	0.126	0.111	0.119	0.014	0.007
9	24000000	60	0.166	0.166	0.102	0.108	0.063	0.059
9	25000000	56	0.132	0.154	0.138	0.116	0.006	0.038
9	26000000	70	0.156	0.144	0.150	0.151	0.006	0.007
9	27000000	78	0.182	0.179	0.177	0.206	0.005	0.027
9	28000000	63	0.145	0.176	0.118	0.137	0.027	0.039
9	29000000	73	0.105	0.115	0.165	0.160	0.060	0.045
9	30000000	64	0.157	0.153	0.146	0.197	0.012	0.044
9	31000000	69	0.159	0.172	0.211	0.210	0.053	0.038
9	32000000	69	0.159	0.179	0.173	0.174	0.014	0.005
9	33000000	59	0.134	0.160	0.105	0.128	0.028	0.032
9	34000000	58	0.209	0.190	0.200	0.218	0.009	0.028
9	35000000	61	0.137	0.127	0.121	0.139	0.015	0.012
9	36000000	49	0.134	0.149	0.160	0.174	0.026	0.025
9	37000000	50	0.106	0.142	0.164	0.173	0.057	0.031
9	38000000	57	0.131	0.175	0.203	0.192	0.072	0.017
9	39000000	65	0.138	0.166	0.176	0.198	0.038	0.033
9	40000000	67	0.147	0.159	0.184	0.233	0.037	0.074
9	41000000	77	0.151	0.120	0.164	0.168	0.013	0.048
9	42000000	67	0.186	0.184	0.171	0.183	0.015	0.001
9	43000000	52	0.133	0.140	0.168	0.158	0.035	0.018
9	44000000	69	0.134	0.123	0.128	0.123	0.006	0.000
9	45000000	59	0.195	0.202	0.147	0.136	0.048	0.066
9	46000000	67	0.186	0.180	0.203	0.223	0.017	0.043
9	47000000	61	0.157	0.177	0.166	0.149	0.009	0.028
9	48000000	85	0.169	0.152	0.145	0.182	0.024	0.030
9	49000000	69	0.184	0.182	0.156	0.167	0.027	0.015
9	50000000	85	0.147	0.159	0.169	0.184	0.022	0.025
9	51000000	86	0.122	0.122	0.146	0.178	0.024	0.057
9	52000000	81	0.177	0.191	0.201	0.191	0.024	0.000
9	53000000	68	0.209	0.196	0.129	0.152	0.080	0.044
9	54000000	66	0.207	0.207	0.197	0.204	0.009	0.003
9	55000000	67	0.226	0.233	0.187	0.205	0.039	0.028
9	56000000	77	0.199	0.188	0.125	0.137	0.074	0.050
9	57000000	51	0.184	0.190	0.114	0.153	0.070	0.036
9	58000000	55	0.178	0.196	0.137	0.136	0.041	0.060
9	59000000	66	0.183	0.155	0.142	0.157	0.041	0.002
9	60000000	71	0.190	0.199	0.174	0.210	0.016	0.012
9	61000000	2	0.150	0.200	0.091	0.112	0.059	0.088
9	84000000	6	0.208	0.186	0.132	0.189	0.076	0.003
10	0	36	0.110	0.108	0.149	0.133	0.039	0.025
10	1000000	46	0.017	0.011	0.067	0.056	0.050	0.045
10	2000000	52	0.021	0.029	0.048	0.053	0.027	0.025
10	3000000	37	0.051	0.051	0.061	0.071	0.010	0.021
10	4000000	53	0.090	0.127	0.149	0.167	0.059	0.040
10	5000000	31	0.060	0.192	0.108	0.132	0.048	0.060
10	6000000	53	0.142	0.141	0.079	0.094	0.063	0.047
10	7000000	58	0.120	0.155	0.101	0.101	0.019	0.054
10	8000000	52	0.013	0.054	0.083	0.078	0.070	0.024
10	9000000	68	0.134	0.145	0.105	0.097	0.029	0.048
10	10000000	69	0.198	0.211	0.163	0.154	0.035	0.057
10	11000000	68	0.160	0.192	0.132	0.143	0.028	0.049
10	12000000	66	0.116	0.165	0.092	0.116	0.024	0.050
10	13000000	60	0.134	0.105	0.085	0.124	0.049	0.019
10	14000000	72	0.139	0.155	0.118	0.143	0.021	0.013
10	15000000	57	0.128	0.168	0.119	0.143	0.009	0.025
10	16000000	36	0.059	0.081	0.067	0.046	0.008	0.035
10	17000000	67	0.142	0.155	0.101	0.129	0.040	0.025
10	18000000	74	0.131	0.149	0.138	0.151	0.006	0.002
10	19000000	84	0.137	0.150	0.117	0.147	0.019	0.002
10	20000000	77	0.119	0.130	0.178	0.162	0.058	0.032
10	21000000	70	0.129	0.147	0.216	0.241	0.087	0.094
10	22000000	78	0.102	0.120	0.170	0.157	0.068	0.037
10	23000000	57	0.057	0.053	0.108	0.122	0.051	0.069
10	24000000	65	0.033	0.028	0.064	0.067	0.031	0.039
10	25000000	75	0.071	0.075	0.113	0.148	0.042	0.073
10	26000000	71	0.189	0.173	0.180	0.211	0.009	0.038
10	27000000	64	0.118	0.110	0.124	0.131	0.006	0.021
10	28000000	66	0.159	0.163	0.164	0.162	0.005	0.001
10	29000000	73	0.134	0.127	0.140	0.152	0.006	0.025
10	30000000	76	0.156	0.142	0.169	0.187	0.013	0.046
10	31000000	67	0.109	0.095	0.139	0.146	0.030	0.051
10	32000000	77	0.161	0.169	0.132	0.176	0.028	0.007
10	33000000	79	0.221	0.192	0.182	0.182	0.039	0.010
10	34000000	63	0.129	0.125	0.117	0.115	0.012	0.010
10	35000000	62	0.118	0.112	0.058	0.065	0.060	0.047
10	36000000	55	0.131	0.148	0.160	0.179	0.029	0.031
10	37000000	75	0.162	0.157	0.167	0.140	0.005	0.017
10	38000000	75	0.189	0.180	0.156	0.172	0.033	0.008
10	39000000	72	0.206	0.208	0.164	0.172	0.041	0.036
10	40000000	69	0.163	0.163	0.155	0.179	0.008	0.016
10	41000000	64	0.177	0.170	0.103	0.111	0.075	0.059
10	42000000	64	0.180	0.183	0.158	0.139	0.021	0.043
10	43000000	68	0.144	0.185	0.149	0.156	0.006	0.030
10	44000000	74	0.128	0.128	0.109	0.118	0.019	0.010
10	45000000	74	0.145	0.144	0.104	0.136	0.041	0.008
10	46000000	61	0.129	0.121	0.081	0.129	0.048	0.007
10	47000000	77	0.169	0.173	0.048	0.101	0.121	0.071
10	48000000	65	0.184	0.184	0.057	0.093	0.127	0.091
10	49000000	72	0.195	0.185	0.123	0.169	0.072	0.016
10	50000000	79	0.155	0.148	0.128	0.152	0.027	0.004
10	51000000	70	0.157	0.164	0.056	0.126	0.101	0.039
10	52000000	59	0.154	0.166	0.130	0.124	0.024	0.043
10	53000000	68	0.166	0.167	0.143	0.119	0.023	0.048
10	54000000	65	0.198	0.183	0.176	0.126	0.023	0.057

10	55000000	73	0.139	0.137	0.153	0.126	0.014	0.011
10	56000000	70	0.197	0.184	0.183	0.209	0.014	0.025
10	57000000	53	0.099	0.114	0.123	0.133	0.024	0.019
10	58000000	64	0.167	0.174	0.155	0.200	0.012	0.026
10	59000000	69	0.100	0.100	0.132	0.151	0.032	0.051
10	60000000	55	0.078	0.107	0.144	0.152	0.066	0.044
10	61000000	61	0.111	0.128	0.147	0.152	0.037	0.024
10	62000000	53	0.135	0.166	0.157	0.144	0.022	0.022
10	63000000	62	0.214	0.192	0.199	0.233	0.015	0.041
10	64000000	66	0.147	0.151	0.148	0.154	0.001	0.003
10	65000000	81	0.151	0.154	0.149	0.143	0.002	0.011
10	66000000	74	0.143	0.170	0.178	0.161	0.035	0.009
10	67000000	71	0.171	0.194	0.220	0.237	0.050	0.042
10	68000000	74	0.160	0.178	0.129	0.135	0.032	0.042
10	69000000	23	0.170	0.179	0.137	0.169	0.033	0.010
10	72000000	2	0.167	0.148	0.104	0.038	0.063	0.109
11	0	26	0.103	0.106	0.100	0.124	0.003	0.018
11	1000000	43	0.053	0.058	0.035	0.045	0.019	0.013
11	2000000	51	0.184	0.104	0.016	0.022	0.169	0.082
11	3000000	47	0.060	0.042	0.044	0.079	0.016	0.037
11	4000000	53	0.129	0.128	0.146	0.148	0.017	0.021
11	5000000	42	0.108	0.104	0.047	0.048	0.061	0.056
11	6000000	55	0.124	0.123	0.072	0.084	0.051	0.038
11	7000000	50	0.143	0.118	0.170	0.148	0.027	0.030
11	8000000	30	0.102	0.154	0.085	0.126	0.017	0.027
11	9000000	20	0.201	0.124	0.142	0.158	0.059	0.034
11	10000000	19	0.216	0.188	0.115	0.080	0.101	0.108
11	11000000	12	0.249	0.231	0.126	0.149	0.123	0.082
11	12000000	60	0.177	0.149	0.141	0.160	0.036	0.012
11	13000000	67	0.108	0.087	0.061	0.074	0.047	0.014
11	14000000	74	0.169	0.163	0.127	0.159	0.043	0.004
11	15000000	70	0.150	0.149	0.138	0.140	0.012	0.010
11	16000000	70	0.197	0.189	0.132	0.164	0.066	0.025
11	17000000	71	0.179	0.182	0.050	0.148	0.130	0.034
11	18000000	69	0.128	0.114	0.123	0.190	0.004	0.075
11	19000000	65	0.085	0.086	0.148	0.177	0.063	0.091
11	20000000	61	0.140	0.126	0.059	0.088	0.081	0.038
11	21000000	68	0.097	0.072	0.084	0.114	0.013	0.043
11	22000000	65	0.141	0.121	0.168	0.157	0.027	0.036
11	23000000	53	0.109	0.102	0.156	0.137	0.047	0.035
11	24000000	56	0.095	0.080	0.043	0.108	0.052	0.028
11	25000000	54	0.054	0.045	0.060	0.087	0.006	0.042
11	26000000	59	0.088	0.065	0.036	0.059	0.053	0.006
11	27000000	49	0.188	0.137	0.118	0.103	0.070	0.034
11	28000000	72	0.134	0.107	0.141	0.119	0.007	0.012
11	29000000	61	0.067	0.052	0.037	0.098	0.029	0.047
11	30000000	50	0.096	0.054	0.135	0.091	0.038	0.037
11	31000000	46	0.141	0.091	0.117	0.107	0.024	0.016
11	32000000	53	0.133	0.093	0.089	0.115	0.043	0.022
11	33000000	71	0.129	0.098	0.138	0.122	0.009	0.023
11	34000000	71	0.157	0.155	0.141	0.130	0.016	0.025
11	35000000	64	0.204	0.228	0.156	0.186	0.048	0.042
11	36000000	66	0.186	0.118	0.041	0.062	0.144	0.056
11	37000000	49	0.107	0.091	0.043	0.057	0.064	0.033
11	38000000	76	0.098	0.074	0.099	0.159	0.001	0.085
11	39000000	75	0.065	0.046	0.099	0.131	0.034	0.085
11	40000000	57	0.050	0.040	0.071	0.115	0.021	0.075
11	41000000	66	0.060	0.049	0.076	0.067	0.016	0.018
11	42000000	70	0.062	0.068	0.063	0.054	0.001	0.014
11	43000000	81	0.081	0.079	0.094	0.089	0.014	0.010
11	44000000	80	0.094	0.097	0.111	0.097	0.017	0.000
11	45000000	68	0.085	0.088	0.188	0.148	0.103	0.060
11	46000000	54	0.066	0.080	0.106	0.086	0.040	0.006
11	47000000	52	0.077	0.084	0.116	0.113	0.040	0.029
11	48000000	49	0.037	0.042	0.120	0.046	0.083	0.004
11	49000000	63	0.074	0.100	0.141	0.115	0.067	0.015
11	50000000	53	0.072	0.106	0.116	0.077	0.044	0.029
11	51000000	51	0.062	0.076	0.068	0.045	0.006	0.030
11	52000000	50	0.103	0.103	0.145	0.116	0.041	0.013
11	53000000	48	0.152	0.187	0.183	0.179	0.030	0.008
11	54000000	71	0.082	0.074	0.074	0.084	0.007	0.010
11	55000000	68	0.183	0.205	0.184	0.185	0.001	0.020
11	56000000	65	0.160	0.163	0.153	0.181	0.007	0.019
11	57000000	68	0.136	0.127	0.101	0.119	0.035	0.008
11	58000000	76	0.122	0.132	0.144	0.155	0.022	0.023
11	59000000	71	0.201	0.204	0.175	0.175	0.026	0.028
11	60000000	83	0.139	0.160	0.154	0.185	0.014	0.024
11	61000000	75	0.101	0.159	0.179	0.201	0.078	0.042
11	62000000	78	0.158	0.179	0.200	0.225	0.042	0.047
11	63000000	89	0.083	0.084	0.129	0.125	0.046	0.041
11	64000000	87	0.074	0.097	0.164	0.165	0.090	0.069
11	65000000	91	0.078	0.099	0.151	0.137	0.073	0.039
11	66000000	72	0.103	0.117	0.141	0.134	0.038	0.018
11	67000000	75	0.137	0.165	0.126	0.165	0.012	0.000
11	68000000	77	0.101	0.110	0.153	0.168	0.053	0.057
11	69000000	78	0.119	0.131	0.151	0.145	0.033	0.014
11	70000000	73	0.153	0.157	0.165	0.193	0.011	0.036
11	71000000	86	0.152	0.159	0.132	0.153	0.021	0.005
11	72000000	75	0.123	0.132	0.145	0.142	0.022	0.010
11	73000000	89	0.132	0.132	0.185	0.181	0.053	0.049
11	74000000	29	0.119	0.121	0.220	0.215	0.101	0.093
11	75000000	1	0.125	0.068	0.083	0.038	0.042	0.031
12	0	73	0.091	0.101	0.106	0.170	0.015	0.069
12	1000000	82	0.102	0.127	0.105	0.171	0.004	0.044
12	2000000	76	0.109	0.115	0.146	0.166	0.036	0.051
12	3000000	71	0.131	0.127	0.129	0.161	0.003	0.033
12	4000000	72	0.149	0.151	0.094	0.129	0.055	0.021
12	5000000	76	0.125	0.140	0.128	0.168	0.003	0.028
12	6000000	62	0.168	0.179	0.144	0.176	0.025	0.003
12	7000000	80	0.193	0.208	0.093	0.137	0.101	0.071
12	8000000	82	0.157	0.145	0.154	0.167	0.003	0.022
12	9000000	76	0.159	0.149	0.165	0.179	0.006	0.030
12	10000000	79	0.161	0.166	0.155	0.174	0.006	0.008
12	11000000	67	0.093	0.118	0.108	0.113	0.015	0.005
12	12000000	77	0.148	0.135	0.150	0.193	0.002	0.059

12	13000000	72	0.136	0.161	0.198	0.197	0.063	0.036
12	14000000	89	0.157	0.160	0.114	0.164	0.044	0.004
12	15000000	92	0.123	0.126	0.121	0.144	0.003	0.018
12	16000000	58	0.197	0.205	0.131	0.133	0.065	0.073
12	17000000	77	0.155	0.173	0.070	0.113	0.085	0.060
12	18000000	78	0.114	0.123	0.084	0.079	0.030	0.044
12	19000000	84	0.147	0.150	0.146	0.130	0.000	0.020
12	20000000	67	0.099	0.131	0.158	0.146	0.059	0.015
12	21000000	72	0.128	0.131	0.140	0.167	0.012	0.035
12	22000000	69	0.095	0.126	0.112	0.151	0.017	0.025
12	23000000	75	0.104	0.114	0.080	0.095	0.024	0.019
12	24000000	58	0.136	0.140	0.092	0.082	0.045	0.058
12	25000000	65	0.152	0.165	0.125	0.114	0.027	0.050
12	26000000	76	0.110	0.073	0.106	0.106	0.004	0.034
12	27000000	71	0.110	0.124	0.100	0.106	0.010	0.018
12	28000000	61	0.137	0.148	0.102	0.084	0.034	0.064
12	29000000	53	0.186	0.214	0.109	0.116	0.076	0.097
12	30000000	53	0.091	0.151	0.161	0.153	0.069	0.002
12	31000000	68	0.108	0.069	0.153	0.101	0.045	0.033
12	32000000	69	0.111	0.100	0.135	0.124	0.024	0.024
12	33000000	61	0.178	0.139	0.075	0.127	0.103	0.012
12	34000000	56	0.113	0.117	0.054	0.072	0.059	0.044
12	35000000	71	0.123	0.128	0.079	0.159	0.044	0.031
12	36000000	66	0.181	0.166	0.180	0.193	0.002	0.027
12	37000000	52	0.163	0.161	0.132	0.156	0.030	0.005
12	38000000	62	0.146	0.179	0.113	0.148	0.033	0.031
12	39000000	68	0.195	0.183	0.136	0.182	0.059	0.001
12	40000000	63	0.187	0.186	0.109	0.122	0.078	0.064
12	41000000	57	0.218	0.190	0.118	0.139	0.100	0.052
12	42000000	69	0.196	0.163	0.136	0.096	0.060	0.067
12	43000000	70	0.174	0.168	0.134	0.177	0.041	0.008
12	44000000	66	0.090	0.100	0.092	0.079	0.001	0.021
12	45000000	68	0.146	0.190	0.097	0.109	0.048	0.081
12	46000000	74	0.218	0.178	0.146	0.160	0.073	0.018
12	47000000	60	0.103	0.102	0.082	0.100	0.021	0.003
12	48000000	59	0.064	0.083	0.122	0.150	0.058	0.066
12	49000000	63	0.175	0.176	0.182	0.209	0.006	0.032
12	50000000	74	0.194	0.194	0.187	0.206	0.008	0.012
12	51000000	76	0.140	0.145	0.212	0.207	0.073	0.062
12	52000000	78	0.125	0.129	0.167	0.209	0.042	0.080
12	53000000	79	0.162	0.175	0.171	0.156	0.009	0.019
12	54000000	70	0.166	0.162	0.146	0.131	0.020	0.032
12	55000000	67	0.146	0.135	0.152	0.140	0.006	0.005
12	56000000	75	0.206	0.201	0.118	0.125	0.088	0.075
12	57000000	85	0.183	0.166	0.137	0.164	0.046	0.002
12	58000000	75	0.145	0.144	0.093	0.111	0.052	0.033
12	59000000	70	0.100	0.101	0.086	0.090	0.014	0.010
12	60000000	75	0.215	0.191	0.114	0.079	0.101	0.112
12	61000000	71	0.159	0.174	0.144	0.171	0.015	0.003
12	62000000	80	0.137	0.123	0.141	0.170	0.004	0.047
12	63000000	69	0.233	0.226	0.166	0.190	0.067	0.036
12	64000000	63	0.196	0.197	0.161	0.183	0.034	0.014
12	65000000	69	0.114	0.128	0.085	0.121	0.030	0.007
12	66000000	61	0.122	0.146	0.136	0.150	0.014	0.003
12	67000000	70	0.127	0.134	0.104	0.127	0.023	0.008
12	68000000	81	0.152	0.140	0.148	0.188	0.004	0.048
12	69000000	74	0.134	0.126	0.116	0.154	0.018	0.028
12	70000000	67	0.157	0.157	0.179	0.198	0.021	0.041
12	71000000	74	0.157	0.137	0.142	0.172	0.015	0.035
12	72000000	39	0.120	0.121	0.111	0.127	0.009	0.006
12	73000000			0.386	0.000	0.163	0.000	0.224
12	74000000	1	0.000	0.000	0.000	0.013	0.000	0.013
13	0	47	0.075	0.169	0.175	0.136	0.100	0.033
13	1000000	61	0.135	0.101	0.148	0.131	0.013	0.030
13	2000000	72	0.181	0.129	0.163	0.206	0.018	0.077
13	3000000	70	0.108	0.082	0.108	0.104	0.000	0.022
13	4000000	79	0.092	0.094	0.073	0.112	0.020	0.018
13	5000000	75	0.101	0.074	0.191	0.135	0.090	0.061
13	6000000	76	0.185	0.183	0.179	0.189	0.006	0.006
13	7000000	74	0.150	0.175	0.184	0.181	0.034	0.005
13	8000000	74	0.142	0.139	0.154	0.148	0.012	0.009
13	9000000	72	0.182	0.177	0.175	0.205	0.008	0.028
13	10000000	70	0.188	0.166	0.185	0.181	0.003	0.015
13	11000000	74	0.113	0.098	0.141	0.151	0.028	0.053
13	12000000	66	0.136	0.123	0.095	0.117	0.041	0.006
13	13000000	76	0.148	0.169	0.182	0.182	0.034	0.013
13	14000000	69	0.151	0.173	0.203	0.177	0.052	0.004
13	15000000	66	0.177	0.200	0.160	0.149	0.017	0.051
13	16000000	73	0.170	0.204	0.139	0.136	0.031	0.068
13	17000000	68	0.105	0.127	0.121	0.146	0.015	0.019
13	18000000	78	0.159	0.156	0.140	0.174	0.020	0.018
13	19000000	79	0.145	0.121	0.131	0.164	0.014	0.043
13	20000000	85	0.142	0.120	0.144	0.184	0.002	0.063
13	21000000	90	0.169	0.135	0.173	0.197	0.004	0.061
13	22000000	76	0.188	0.186	0.131	0.189	0.057	0.003
13	23000000	74	0.162	0.174	0.117	0.159	0.044	0.015
13	24000000	76	0.181	0.180	0.150	0.183	0.032	0.003
13	25000000	76	0.163	0.158	0.108	0.153	0.055	0.005
13	26000000	51	0.096	0.094	0.116	0.141	0.020	0.047
13	27000000	80	0.149	0.128	0.152	0.180	0.003	0.052
13	28000000	93	0.137	0.099	0.170	0.165	0.033	0.065
13	29000000	95	0.152	0.120	0.166	0.183	0.014	0.063
13	30000000	70	0.141	0.132	0.165	0.175	0.024	0.043
13	31000000	78	0.169	0.187	0.203	0.238	0.035	0.050
13	32000000	71	0.147	0.141	0.196	0.228	0.049	0.087
13	33000000	94	0.115	0.102	0.120	0.152	0.005	0.050
13	34000000	87	0.128	0.115	0.224	0.231	0.096	0.116
13	35000000	76	0.147	0.112	0.224	0.183	0.077	0.070
13	36000000	60	0.180	0.164	0.186	0.182	0.006	0.019
13	37000000	60	0.121	0.141	0.186	0.188	0.065	0.046
13	38000000	50	0.192	0.248	0.173	0.216	0.019	0.032
13	39000000	63	0.125	0.160	0.161	0.168	0.036	0.009
13	40000000	68	0.126	0.138	0.141	0.186	0.015	0.048
13	41000000	57	0.187	0.185	0.203	0.210	0.016	0.024
13	42000000	75	0.080	0.083	0.145	0.131	0.065	0.048

13	43000000	75	0.179	0.175	0.177	0.164	0.002	0.012
13	44000000	66	0.224	0.254	0.198	0.205	0.027	0.049
13	45000000	73	0.110	0.127	0.150	0.176	0.040	0.050
13	46000000	73	0.152	0.161	0.169	0.195	0.017	0.034
13	47000000	81	0.137	0.142	0.124	0.129	0.012	0.012
13	48000000	60	0.068	0.055	0.103	0.098	0.035	0.043
13	49000000	66	0.134	0.144	0.187	0.195	0.054	0.051
13	50000000	60	0.149	0.158	0.212	0.224	0.063	0.066
13	51000000	64	0.139	0.190	0.153	0.172	0.013	0.018
13	52000000	66	0.135	0.180	0.156	0.142	0.021	0.038
13	53000000	68	0.161	0.197	0.179	0.179	0.018	0.018
13	54000000	64	0.111	0.109	0.164	0.155	0.053	0.047
13	55000000	60	0.147	0.171	0.167	0.182	0.021	0.011
13	56000000	68	0.139	0.157	0.211	0.234	0.071	0.077
13	57000000	69	0.161	0.160	0.205	0.199	0.044	0.039
13	58000000	70	0.205	0.206	0.170	0.192	0.035	0.013
13	59000000	75	0.190	0.197	0.170	0.193	0.021	0.003
13	60000000	83	0.149	0.160	0.138	0.148	0.010	0.011
13	61000000	75	0.166	0.154	0.147	0.200	0.019	0.047
13	62000000	75	0.157	0.164	0.143	0.191	0.014	0.027
13	63000000	13	0.152	0.160	0.311	0.288	0.159	0.128
13	65000000	1	0.375	0.364	0.417	0.388	0.042	0.024
14	0	39	0.103	0.101	0.077	0.079	0.026	0.022
14	1000000	38	0.117	0.127	0.113	0.082	0.004	0.045
14	2000000	41	0.144	0.162	0.190	0.172	0.046	0.010
14	3000000	88	0.176	0.221	0.218	0.149	0.042	0.072
14	4000000	67	0.139	0.145	0.133	0.126	0.005	0.019
14	5000000	62	0.114	0.085	0.120	0.125	0.006	0.040
14	6000000	43	0.128	0.093	0.092	0.062	0.036	0.031
14	7000000	60	0.144	0.137	0.069	0.070	0.076	0.067
14	8000000	72	0.168	0.154	0.071	0.093	0.097	0.061
14	9000000	67	0.163	0.181	0.225	0.176	0.062	0.005
14	10000000	72	0.188	0.178	0.113	0.182	0.075	0.003
14	11000000	59	0.172	0.189	0.158	0.184	0.014	0.005
14	12000000	65	0.169	0.189	0.186	0.186	0.017	0.003
14	13000000	83	0.126	0.122	0.128	0.124	0.002	0.002
14	14000000	72	0.109	0.096	0.129	0.107	0.020	0.012
14	15000000	79	0.133	0.106	0.165	0.191	0.033	0.085
14	16000000	69	0.233	0.192	0.174	0.190	0.059	0.003
14	17000000	74	0.132	0.113	0.121	0.185	0.011	0.072
14	18000000	61	0.078	0.107	0.150	0.170	0.072	0.063
14	19000000	52	0.107	0.126	0.149	0.146	0.042	0.020
14	20000000	73	0.140	0.147	0.124	0.165	0.017	0.018
14	21000000	62	0.132	0.135	0.112	0.140	0.021	0.005
14	22000000	80	0.162	0.166	0.192	0.194	0.030	0.029
14	23000000	72	0.089	0.106	0.209	0.174	0.119	0.068
14	24000000	73	0.105	0.098	0.180	0.203	0.075	0.105
14	25000000	74	0.104	0.109	0.209	0.184	0.104	0.075
14	26000000	73	0.137	0.161	0.224	0.220	0.087	0.060
14	27000000	77	0.138	0.111	0.183	0.168	0.044	0.056
14	28000000	60	0.129	0.130	0.183	0.205	0.053	0.075
14	29000000	78	0.195	0.177	0.187	0.202	0.009	0.025
14	30000000	68	0.135	0.119	0.160	0.140	0.026	0.020
14	31000000	71	0.192	0.174	0.217	0.178	0.025	0.004
14	32000000	71	0.153	0.132	0.145	0.137	0.008	0.005
14	33000000	68	0.146	0.106	0.168	0.171	0.023	0.065
14	34000000	61	0.128	0.105	0.112	0.122	0.017	0.017
14	35000000	81	0.177	0.179	0.178	0.170	0.001	0.009
14	36000000	77	0.165	0.189	0.175	0.197	0.010	0.009
14	37000000	64	0.159	0.118	0.180	0.203	0.021	0.085
14	38000000	79	0.163	0.153	0.161	0.164	0.001	0.011
14	39000000	73	0.198	0.201	0.125	0.125	0.073	0.076
14	40000000	75	0.166	0.147	0.138	0.134	0.028	0.013
14	41000000	73	0.166	0.156	0.095	0.111	0.071	0.045
14	42000000	68	0.174	0.173	0.164	0.172	0.010	0.001
14	43000000	80	0.084	0.099	0.148	0.126	0.064	0.026
14	44000000	74	0.157	0.170	0.167	0.163	0.011	0.008
14	45000000	57	0.075	0.082	0.104	0.116	0.029	0.034
14	46000000	75	0.117	0.113	0.099	0.113	0.019	0.000
14	47000000	68	0.185	0.208	0.184	0.202	0.000	0.006
14	48000000	68	0.160	0.170	0.142	0.185	0.019	0.015
14	49000000	62	0.159	0.167	0.134	0.179	0.025	0.012
14	50000000	72	0.136	0.136	0.138	0.165	0.002	0.030
14	51000000	66	0.119	0.116	0.074	0.127	0.045	0.011
14	52000000	55	0.152	0.185	0.126	0.125	0.027	0.061
14	53000000	59	0.138	0.137	0.099	0.106	0.039	0.031
14	54000000	67	0.162	0.190	0.152	0.168	0.011	0.021
14	55000000	62	0.081	0.110	0.100	0.095	0.019	0.015
14	56000000	75	0.119	0.147	0.147	0.172	0.028	0.025
14	57000000	58	0.150	0.171	0.175	0.186	0.025	0.015
14	58000000	78	0.148	0.169	0.161	0.173	0.013	0.004
14	59000000	77	0.146	0.146	0.154	0.179	0.008	0.033
14	60000000	80	0.124	0.092	0.111	0.115	0.013	0.023
14	63000000	1	0.000	0.000	0.000	0.000	0.000	0.000
15	0	38	0.192	0.075	0.078	0.117	0.114	0.041
15	1000000	51	0.111	0.082	0.065	0.063	0.046	0.019
15	2000000	46	0.085	0.065	0.052	0.082	0.032	0.017
15	3000000	61	0.106	0.071	0.091	0.104	0.015	0.034
15	4000000	55	0.123	0.146	0.125	0.155	0.002	0.009
15	5000000	53	0.038	0.032	0.057	0.059	0.020	0.026
15	6000000	63	0.161	0.063	0.077	0.112	0.084	0.049
15	7000000	64	0.176	0.143	0.123	0.141	0.053	0.001
15	8000000	55	0.090	0.111	0.124	0.143	0.033	0.032
15	9000000	66	0.161	0.112	0.110	0.168	0.051	0.056
15	10000000	51	0.075	0.079	0.075	0.093	0.000	0.014
15	11000000	45	0.038	0.047	0.062	0.085	0.025	0.038
15	12000000	73	0.133	0.140	0.131	0.143	0.002	0.003
15	13000000	68	0.170	0.168	0.119	0.172	0.051	0.004
15	14000000	50	0.159	0.176	0.146	0.145	0.013	0.031
15	15000000	70	0.191	0.163	0.171	0.194	0.020	0.031
15	16000000	75	0.127	0.118	0.104	0.146	0.023	0.028
15	17000000	52	0.123	0.145	0.112	0.131	0.010	0.014
15	18000000	66	0.179	0.196	0.152	0.181	0.027	0.015
15	19000000	67	0.171	0.191	0.126	0.160	0.045	0.032
15	20000000	81	0.153	0.167	0.103	0.135	0.050	0.031

15	21000000	78	0.217	0.219	0.157	0.190	0.061	0.029
15	22000000	75	0.173	0.193	0.145	0.206	0.028	0.013
15	23000000	71	0.171	0.199	0.170	0.188	0.002	0.011
15	24000000	74	0.172	0.186	0.185	0.190	0.013	0.005
15	25000000	72	0.167	0.136	0.107	0.159	0.060	0.023
15	26000000	67	0.155	0.137	0.157	0.159	0.002	0.022
15	27000000	79	0.192	0.138	0.098	0.123	0.095	0.015
15	28000000	81	0.188	0.133	0.130	0.170	0.058	0.037
15	29000000	69	0.074	0.055	0.109	0.158	0.036	0.103
15	30000000	76	0.141	0.116	0.144	0.149	0.003	0.033
15	31000000	70	0.150	0.136	0.147	0.162	0.003	0.025
15	32000000	54	0.160	0.185	0.193	0.239	0.033	0.053
15	33000000	59	0.111	0.155	0.078	0.108	0.033	0.048
15	34000000	74	0.127	0.090	0.073	0.078	0.054	0.012
15	35000000	60	0.130	0.103	0.068	0.053	0.061	0.050
15	36000000	62	0.179	0.150	0.176	0.183	0.003	0.033
15	37000000	62	0.178	0.163	0.119	0.127	0.059	0.036
15	38000000	55	0.128	0.150	0.115	0.106	0.013	0.044
15	39000000	62	0.133	0.165	0.101	0.120	0.033	0.045
15	40000000	65	0.180	0.146	0.139	0.151	0.041	0.004
15	41000000	61	0.154	0.179	0.100	0.115	0.055	0.064
15	42000000	66	0.176	0.213	0.176	0.180	0.001	0.033
15	43000000	70	0.228	0.232	0.174	0.184	0.054	0.048
15	44000000	69	0.201	0.204	0.115	0.118	0.086	0.086
15	45000000	73	0.221	0.224	0.070	0.195	0.151	0.029
15	46000000	50	0.195	0.210	0.031	0.106	0.164	0.104
15	47000000	72	0.147	0.145	0.141	0.165	0.006	0.020
15	48000000	67	0.183	0.194	0.114	0.144	0.069	0.049
15	49000000	71	0.194	0.189	0.174	0.189	0.020	0.000
15	50000000	57	0.136	0.182	0.151	0.172	0.015	0.009
15	51000000	64	0.177	0.170	0.197	0.210	0.020	0.040
15	52000000	75	0.154	0.154	0.179	0.188	0.025	0.033
15	53000000	82	0.140	0.129	0.189	0.194	0.049	0.066
15	54000000	67	0.138	0.156	0.226	0.215	0.088	0.059
15	55000000	72	0.216	0.234	0.161	0.197	0.055	0.037
15	56000000	79	0.171	0.199	0.192	0.200	0.021	0.001
15	57000000	62	0.196	0.208	0.111	0.136	0.085	0.072
15	58000000	66	0.165	0.144	0.155	0.205	0.010	0.061
15	59000000	76	0.196	0.181	0.192	0.185	0.004	0.005
15	60000000	71	0.213	0.203	0.196	0.221	0.017	0.018
15	61000000	66	0.166	0.162	0.162	0.164	0.004	0.002
15	62000000	73	0.156	0.130	0.210	0.209	0.054	0.079
15	63000000	58	0.168	0.182	0.178	0.188	0.010	0.005
15	64000000	16	0.117	0.155	0.120	0.114	0.003	0.041
16	0	45	0.180	0.173	0.166	0.132	0.014	0.041
16	1000000	27	0.140	0.132	0.072	0.087	0.069	0.045
16	2000000	60	0.110	0.095	0.089	0.095	0.021	0.000
16	3000000	74	0.191	0.152	0.136	0.158	0.055	0.006
16	4000000	54	0.170	0.168	0.157	0.148	0.013	0.020
16	5000000	54	0.145	0.139	0.212	0.151	0.068	0.013
16	6000000	65	0.196	0.200	0.152	0.138	0.044	0.062
16	7000000	67	0.137	0.154	0.140	0.141	0.003	0.013
16	8000000	64	0.129	0.138	0.167	0.197	0.038	0.059
16	9000000	51	0.064	0.045	0.061	0.059	0.003	0.014
16	10000000	37	0.099	0.085	0.148	0.166	0.049	0.081
16	11000000	80	0.121	0.095	0.130	0.161	0.009	0.066
16	12000000	69	0.183	0.183	0.177	0.131	0.007	0.052
16	13000000	51	0.108	0.139	0.098	0.116	0.010	0.023
16	14000000	63	0.166	0.175	0.138	0.160	0.029	0.015
16	15000000	72	0.191	0.191	0.101	0.146	0.089	0.044
16	16000000	63	0.163	0.152	0.101	0.132	0.062	0.020
16	17000000	97	0.154	0.134	0.124	0.140	0.030	0.006
16	18000000	74	0.147	0.159	0.118	0.139	0.028	0.020
16	19000000	79	0.184	0.197	0.169	0.177	0.015	0.020
16	20000000	68	0.124	0.106	0.138	0.181	0.014	0.075
16	21000000	60	0.115	0.081	0.135	0.172	0.019	0.092
16	22000000	61	0.148	0.145	0.144	0.175	0.004	0.031
16	23000000	66	0.153	0.112	0.152	0.145	0.002	0.033
16	24000000	73	0.132	0.119	0.144	0.153	0.012	0.034
16	25000000	51	0.149	0.166	0.148	0.171	0.000	0.005
16	26000000	48	0.144	0.120	0.115	0.135	0.029	0.015
16	27000000	73	0.167	0.128	0.116	0.166	0.051	0.038
16	28000000	66	0.193	0.148	0.140	0.135	0.052	0.013
16	29000000	62	0.131	0.113	0.158	0.181	0.027	0.067
16	30000000	76	0.164	0.162	0.123	0.138	0.042	0.024
16	31000000	78	0.168	0.150	0.109	0.153	0.059	0.004
16	32000000	70	0.181	0.164	0.112	0.162	0.069	0.003
16	33000000	63	0.153	0.151	0.048	0.078	0.106	0.074
16	34000000	59	0.111	0.110	0.088	0.092	0.023	0.017
16	35000000	76	0.169	0.165	0.151	0.181	0.017	0.016
16	36000000	84	0.116	0.133	0.123	0.140	0.007	0.007
16	37000000	70	0.102	0.135	0.133	0.169	0.031	0.034
16	38000000	64	0.206	0.221	0.150	0.178	0.056	0.043
16	39000000	62	0.161	0.132	0.102	0.136	0.060	0.004
16	40000000	74	0.122	0.120	0.081	0.129	0.041	0.009
16	41000000	73	0.170	0.156	0.136	0.135	0.034	0.022
16	42000000	74	0.207	0.208	0.201	0.207	0.006	0.001
16	43000000	74	0.167	0.141	0.149	0.172	0.018	0.031
16	44000000	70	0.145	0.153	0.165	0.158	0.020	0.004
16	45000000	76	0.205	0.165	0.128	0.172	0.077	0.006
16	46000000	82	0.183	0.180	0.116	0.162	0.067	0.018
16	47000000	67	0.195	0.210	0.130	0.188	0.065	0.022
16	48000000	75	0.190	0.183	0.154	0.154	0.037	0.029
16	49000000	73	0.149	0.152	0.140	0.164	0.009	0.012
16	50000000	68	0.138	0.156	0.156	0.179	0.018	0.023
16	51000000	59	0.177	0.181	0.140	0.149	0.037	0.031
16	52000000	71	0.193	0.192	0.131	0.160	0.062	0.032
16	53000000	73	0.129	0.141	0.145	0.144	0.016	0.002
16	54000000	65	0.168	0.129	0.182	0.178	0.014	0.048
16	55000000	84	0.110	0.092	0.185	0.171	0.074	0.080
16	56000000	77	0.095	0.067	0.141	0.148	0.046	0.081
16	57000000	74	0.104	0.071	0.094	0.140	0.009	0.069
16	58000000	71	0.108	0.035	0.187	0.176	0.079	0.141
16	59000000	5	0.017	0.009	0.192	0.067	0.175	0.058
		1	0.417		0.333	0.275	0.083	0.275

17	0	58	0.145	0.166	0.123	0.100	0.022	0.066
17	1000000	67	0.085	0.100	0.108	0.142	0.023	0.042
17	2000000	73	0.164	0.115	0.061	0.126	0.102	0.012
17	3000000	87	0.231	0.172	0.248	0.300	0.018	0.128
17	4000000	69	0.175	0.182	0.282	0.277	0.107	0.094
17	5000000	74	0.223	0.205	0.245	0.188	0.022	0.016
17	6000000	46	0.185	0.189	0.179	0.177	0.006	0.012
17	7000000	75	0.062	0.093	0.131	0.141	0.069	0.047
17	8000000	87	0.075	0.111	0.149	0.165	0.074	0.054
17	9000000	88	0.095	0.106	0.172	0.211	0.076	0.105
17	10000000	81	0.077	0.114	0.159	0.178	0.082	0.064
17	11000000	81	0.079	0.096	0.169	0.170	0.090	0.074
17	12000000	81	0.161	0.186	0.186	0.199	0.025	0.013
17	13000000	76	0.104	0.146	0.150	0.176	0.045	0.030
17	14000000	73	0.180	0.183	0.150	0.173	0.030	0.010
17	15000000	68	0.185	0.180	0.194	0.184	0.009	0.003
17	16000000	67	0.140	0.136	0.157	0.148	0.017	0.012
17	17000000	70	0.126	0.141	0.143	0.177	0.017	0.035
17	18000000	74	0.183	0.179	0.196	0.208	0.012	0.029
17	19000000	61	0.077	0.070	0.126	0.153	0.049	0.083
17	20000000	76	0.110	0.094	0.166	0.154	0.056	0.060
17	21000000	46	0.058	0.055	0.077	0.111	0.019	0.056
17	22000000	77	0.164	0.159	0.137	0.165	0.026	0.006
17	23000000	83	0.133	0.167	0.180	0.195	0.047	0.028
17	24000000	77	0.174	0.183	0.185	0.195	0.010	0.012
17	25000000	66	0.177	0.185	0.156	0.199	0.021	0.014
17	26000000	71	0.197	0.213	0.195	0.232	0.002	0.019
17	27000000	61	0.161	0.179	0.178	0.190	0.017	0.010
17	28000000	79	0.180	0.166	0.181	0.202	0.001	0.036
17	29000000	64	0.131	0.103	0.151	0.154	0.020	0.051
17	30000000	67	0.143	0.118	0.135	0.127	0.007	0.009
17	31000000	71	0.151	0.139	0.153	0.170	0.002	0.031
17	32000000	77	0.151	0.139	0.155	0.184	0.004	0.045
17	33000000	81	0.198	0.171	0.171	0.183	0.028	0.012
17	34000000	77	0.166	0.167	0.175	0.149	0.009	0.017
17	35000000	69	0.158	0.154	0.183	0.166	0.025	0.012
17	36000000	70	0.185	0.169	0.140	0.142	0.045	0.027
17	37000000	68	0.134	0.143	0.090	0.090	0.044	0.053
17	38000000	62	0.132	0.135	0.162	0.152	0.029	0.017
17	39000000	72	0.184	0.179	0.170	0.168	0.014	0.012
17	40000000	76	0.165	0.139	0.160	0.125	0.005	0.014
17	41000000	67	0.158	0.159	0.195	0.175	0.038	0.016
17	42000000	71	0.171	0.164	0.126	0.124	0.045	0.040
17	43000000	64	0.171	0.180	0.157	0.134	0.014	0.046
17	44000000	68	0.170	0.192	0.170	0.156	0.000	0.036
17	45000000	61	0.212	0.207	0.193	0.194	0.019	0.013
17	46000000	77	0.128	0.138	0.151	0.137	0.023	0.001
17	47000000	76	0.183	0.195	0.149	0.179	0.034	0.016
17	48000000	92	0.140	0.129	0.172	0.206	0.032	0.077
17	49000000	89	0.178	0.187	0.188	0.200	0.010	0.013
17	50000000	68	0.190	0.199	0.203	0.179	0.012	0.021
17	51000000	86	0.172	0.205	0.182	0.190	0.010	0.015
17	52000000	67	0.196	0.201	0.210	0.223	0.014	0.022
17	53000000	77	0.192	0.171	0.185	0.194	0.007	0.022
17	54000000	82	0.202	0.199	0.176	0.198	0.026	0.002
17	55000000	64	0.142	0.131	0.178	0.181	0.036	0.050
17	56000000	57	0.178	0.181	0.184	0.191	0.006	0.010
17	57000000	44	0.119	0.100	0.124	0.115	0.005	0.015
17	58000000	53	0.145	0.150	0.127	0.105	0.018	0.045
17	59000000	62	0.087	0.117	0.115	0.127	0.028	0.010
17	60000000	72	0.143	0.181	0.166	0.162	0.023	0.019
17	61000000	47	0.172	0.174	0.161	0.170	0.012	0.004
17	62000000	65	0.188	0.192	0.147	0.171	0.041	0.020
17	63000000	72	0.168	0.170	0.119	0.118	0.049	0.052
17	64000000	6	0.079	0.099	0.056	0.071	0.023	0.028
18	0	53	0.167	0.188	0.190	0.170	0.023	0.018
18	1000000	55	0.050	0.058	0.078	0.065	0.029	0.007
18	2000000	74	0.187	0.167	0.109	0.123	0.078	0.044
18	3000000	39	0.091	0.131	0.069	0.167	0.022	0.036
18	4000000	53	0.068	0.069	0.067	0.065	0.001	0.004
18	5000000	69	0.076	0.095	0.137	0.078	0.061	0.016
18	6000000	72	0.225	0.254	0.231	0.227	0.006	0.027
18	7000000	66	0.184	0.188	0.155	0.166	0.029	0.022
18	8000000	72	0.115	0.128	0.177	0.160	0.062	0.032
18	9000000	57	0.165	0.177	0.123	0.127	0.043	0.050
18	10000000	75	0.169	0.131	0.146	0.188	0.022	0.057
18	11000000	61	0.202	0.166	0.141	0.164	0.061	0.002
18	12000000	77	0.092	0.098	0.035	0.059	0.058	0.038
18	13000000	73	0.138	0.146	0.139	0.142	0.001	0.004
18	14000000	70	0.199	0.204	0.180	0.189	0.020	0.015
18	15000000	74	0.124	0.113	0.105	0.112	0.020	0.001
18	16000000	79	0.194	0.161	0.149	0.160	0.045	0.001
18	17000000	59	0.227	0.253	0.197	0.237	0.030	0.017
18	18000000	38	0.229	0.258	0.159	0.199	0.071	0.059
18	19000000	56	0.174	0.155	0.121	0.153	0.054	0.002
18	20000000	57	0.092	0.100	0.133	0.152	0.041	0.051
18	21000000	74	0.156	0.164	0.137	0.161	0.020	0.004
18	22000000	76	0.167	0.164	0.128	0.158	0.039	0.006
18	23000000	76	0.161	0.157	0.127	0.141	0.034	0.015
18	24000000	64	0.118	0.134	0.119	0.140	0.001	0.007
18	25000000	51	0.180	0.195	0.205	0.236	0.025	0.042
18	26000000	79	0.167	0.144	0.117	0.145	0.050	0.001
18	27000000	72	0.144	0.153	0.153	0.164	0.009	0.011
18	28000000	83	0.150	0.146	0.143	0.169	0.007	0.023
18	29000000	69	0.093	0.090	0.158	0.172	0.065	0.082
18	30000000	83	0.161	0.127	0.181	0.177	0.020	0.050
18	31000000	92	0.124	0.110	0.152	0.173	0.028	0.062
18	32000000	81	0.158	0.142	0.154	0.172	0.004	0.029
18	33000000	74	0.183	0.182	0.194	0.225	0.012	0.044
18	34000000	74	0.199	0.176	0.192	0.211	0.007	0.035
18	35000000	88	0.139	0.125	0.171	0.193	0.032	0.068
18	36000000	90	0.105	0.106	0.185	0.189	0.080	0.082
18	37000000	66	0.159	0.169	0.172	0.184	0.013	0.015
18	38000000	79	0.139	0.133	0.125	0.163	0.014	0.030
18	39000000	64	0.145	0.147	0.168	0.176	0.023	0.029

18	40000000	38	0.161	0.167	0.139	0.183	0.023	0.016
18	41000000	56	0.120	0.103	0.155	0.188	0.036	0.085
18	42000000	76	0.108	0.092	0.105	0.171	0.003	0.079
18	43000000	80	0.076	0.087	0.120	0.131	0.044	0.044
18	44000000	81	0.142	0.141	0.134	0.163	0.008	0.021
18	45000000	71	0.139	0.132	0.147	0.174	0.008	0.041
18	46000000	88	0.142	0.115	0.137	0.145	0.005	0.030
18	47000000	85	0.140	0.112	0.128	0.151	0.012	0.039
18	48000000	78	0.163	0.140	0.145	0.179	0.018	0.039
18	49000000	74	0.178	0.178	0.149	0.178	0.029	0.000
18	50000000	70	0.095	0.114	0.155	0.192	0.060	0.077
18	51000000	74	0.154	0.156	0.159	0.152	0.004	0.003
18	52000000	82	0.122	0.120	0.164	0.148	0.042	0.029
18	53000000	70	0.108	0.106	0.163	0.149	0.056	0.043
18	54000000	84	0.149	0.150	0.219	0.246	0.070	0.096
18	55000000	57	0.179	0.187	0.231	0.198	0.052	0.011
18	57000000	1	0.125	0.136	0.059	0.089	0.066	0.047
19	0	55	0.080	0.075	0.121	0.120	0.041	0.045
19	1000000	67	0.124	0.098	0.061	0.072	0.063	0.026
19	2000000	58	0.027	0.018	0.047	0.052	0.019	0.034
19	3000000	54	0.050	0.049	0.131	0.150	0.080	0.101
19	4000000	86	0.220	0.195	0.151	0.162	0.069	0.033
19	5000000	49	0.155	0.165	0.148	0.143	0.006	0.022
19	6000000	60	0.250	0.284	0.187	0.195	0.063	0.089
19	7000000	63	0.239	0.235	0.191	0.196	0.048	0.039
19	8000000	51	0.204	0.191	0.124	0.151	0.080	0.040
19	9000000	67	0.184	0.160	0.146	0.163	0.038	0.003
19	10000000	61	0.210	0.184	0.140	0.179	0.069	0.005
19	11000000	67	0.168	0.135	0.153	0.134	0.015	0.001
19	12000000	59	0.124	0.092	0.067	0.085	0.057	0.007
19	13000000	61	0.090	0.094	0.119	0.125	0.029	0.031
19	14000000	66	0.111	0.142	0.125	0.150	0.015	0.008
19	15000000	78	0.178	0.188	0.223	0.217	0.046	0.028
19	16000000	84	0.188	0.187	0.148	0.164	0.040	0.023
19	17000000	52	0.144	0.184	0.171	0.175	0.027	0.009
19	18000000	66	0.180	0.196	0.146	0.164	0.034	0.032
19	19000000	52	0.151	0.149	0.154	0.122	0.003	0.027
19	20000000	59	0.123	0.127	0.152	0.165	0.028	0.038
19	21000000	67	0.195	0.202	0.109	0.129	0.086	0.073
19	22000000	63	0.153	0.164	0.111	0.154	0.042	0.010
19	23000000	68	0.185	0.195	0.158	0.173	0.028	0.021
19	24000000	67	0.185	0.186	0.150	0.166	0.035	0.020
19	25000000	70	0.183	0.176	0.167	0.188	0.017	0.012
19	26000000	75	0.165	0.137	0.156	0.178	0.009	0.041
19	27000000	80	0.160	0.128	0.200	0.245	0.040	0.116
19	28000000	82	0.164	0.122	0.129	0.172	0.035	0.050
19	29000000	83	0.136	0.118	0.187	0.208	0.051	0.089
19	30000000	77	0.207	0.171	0.123	0.149	0.084	0.022
19	31000000	95	0.135	0.129	0.187	0.214	0.052	0.086
19	32000000	76	0.168	0.157	0.169	0.208	0.001	0.051
19	33000000	83	0.156	0.135	0.139	0.167	0.017	0.033
19	34000000	82	0.125	0.106	0.151	0.174	0.027	0.068
19	35000000	84	0.160	0.152	0.169	0.179	0.009	0.028
19	36000000	75	0.143	0.131	0.132	0.164	0.010	0.033
19	37000000	75	0.102	0.092	0.138	0.146	0.036	0.054
19	38000000	64	0.063	0.073	0.171	0.185	0.108	0.112
19	39000000	70	0.169	0.183	0.154	0.183	0.015	0.001
19	40000000	69	0.191	0.151	0.143	0.180	0.048	0.028
19	41000000	66	0.126	0.138	0.167	0.206	0.040	0.068
19	42000000	78	0.173	0.128	0.142	0.164	0.032	0.037
19	43000000	66	0.140	0.122	0.159	0.175	0.018	0.053
19	44000000	73	0.140	0.102	0.170	0.194	0.030	0.091
19	45000000	79	0.135	0.078	0.200	0.185	0.065	0.107
19	46000000	83	0.145	0.147	0.179	0.187	0.034	0.040
19	47000000	83	0.133	0.132	0.206	0.181	0.073	0.049
19	48000000	81	0.190	0.152	0.176	0.176	0.015	0.025
19	49000000	67	0.148	0.126	0.132	0.146	0.017	0.021
19	50000000	66	0.166	0.167	0.157	0.165	0.009	0.002
19	51000000	74	0.134	0.125	0.206	0.193	0.072	0.069
19	52000000	63	0.142	0.127	0.189	0.208	0.048	0.081
19	53000000	47	0.160	0.169	0.098	0.120	0.062	0.049
20	0	48	0.162	0.167	0.066	0.089	0.096	0.077
20	1000000	54	0.030	0.050	0.104	0.127	0.074	0.077
20	2000000	60	0.039	0.073	0.080	0.164	0.042	0.091
20	3000000	78	0.127	0.070	0.081	0.107	0.046	0.036
20	4000000	77	0.092	0.062	0.119	0.113	0.026	0.051
20	5000000	66	0.140	0.123	0.131	0.131	0.008	0.008
20	6000000	61	0.090	0.133	0.124	0.162	0.034	0.029
20	7000000	67	0.081	0.093	0.146	0.172	0.065	0.079
20	8000000	65	0.128	0.105	0.124	0.142	0.003	0.038
20	9000000	77	0.095	0.091	0.141	0.149	0.047	0.058
20	10000000	73	0.164	0.171	0.086	0.096	0.078	0.075
20	11000000	67	0.117	0.110	0.131	0.126	0.015	0.016
20	12000000	75	0.153	0.119	0.171	0.178	0.018	0.059
20	13000000	72	0.146	0.126	0.122	0.093	0.024	0.033
20	14000000	65	0.190	0.173	0.104	0.102	0.086	0.071
20	15000000	65	0.180	0.187	0.138	0.155	0.042	0.032
20	16000000	67	0.178	0.177	0.207	0.226	0.028	0.049
20	17000000	73	0.246	0.240	0.153	0.155	0.093	0.085
20	18000000	77	0.171	0.167	0.144	0.127	0.027	0.040
20	19000000	77	0.166	0.164	0.165	0.147	0.001	0.017
20	20000000	76	0.120	0.169	0.117	0.114	0.003	0.055
20	21000000	56	0.032	0.122	0.112	0.110	0.080	0.012
20	22000000	84	0.142	0.130	0.132	0.142	0.010	0.013
20	23000000	73	0.077	0.066	0.130	0.150	0.053	0.084
20	24000000	82	0.083	0.086	0.140	0.190	0.057	0.104
20	25000000	69	0.173	0.181	0.158	0.168	0.015	0.013
20	26000000	65	0.154	0.159	0.162	0.168	0.009	0.008
20	27000000	64	0.174	0.197	0.188	0.203	0.015	0.007
20	28000000	71	0.156	0.160	0.139	0.149	0.017	0.011
20	29000000	69	0.144	0.141	0.123	0.136	0.022	0.005
20	30000000	58	0.152	0.135	0.167	0.178	0.015	0.043
20	31000000	73	0.136	0.127	0.178	0.174	0.042	0.048
20	32000000	63	0.157	0.126	0.141	0.164	0.016	0.038
20	33000000	70	0.151	0.167	0.184	0.230	0.033	0.063

20	34000000	68	0.172	0.127	0.149	0.168	0.023	0.040
20	35000000	75	0.161	0.139	0.109	0.129	0.052	0.010
20	36000000	66	0.119	0.109	0.104	0.128	0.015	0.019
20	37000000	78	0.130	0.107	0.090	0.111	0.040	0.003
20	38000000	70	0.163	0.148	0.125	0.116	0.038	0.032
20	39000000	59	0.139	0.118	0.106	0.148	0.033	0.029
20	40000000	57	0.148	0.136	0.072	0.061	0.076	0.075
20	41000000	49	0.063	0.082	0.156	0.189	0.093	0.107
20	42000000	74	0.167	0.152	0.144	0.113	0.023	0.039
20	43000000	75	0.144	0.139	0.130	0.133	0.014	0.006
20	44000000	61	0.139	0.169	0.148	0.175	0.010	0.006
20	45000000	69	0.182	0.177	0.136	0.176	0.046	0.001
20	46000000	66	0.168	0.156	0.152	0.198	0.016	0.042
20	47000000	50	0.099	0.126	0.094	0.116	0.005	0.010
20	48000000	71	0.118	0.110	0.077	0.106	0.041	0.004
20	49000000	65	0.069	0.060	0.148	0.168	0.080	0.108
20	50000000	81	0.140	0.115	0.127	0.142	0.012	0.027
20	51000000	76	0.113	0.119	0.165	0.188	0.052	0.068
20	52000000	78	0.162	0.136	0.165	0.191	0.003	0.055
20	53000000	67	0.105	0.106	0.173	0.202	0.067	0.096
20	54000000	87	0.148	0.147	0.143	0.163	0.005	0.017
20	55000000	62	0.197	0.203	0.179	0.155	0.019	0.048
20	56000000	73	0.113	0.106	0.165	0.182	0.052	0.076
20	57000000	70	0.125	0.145	0.233	0.275	0.108	0.130
20	58000000	5	0.208	0.313	0.207	0.162	0.001	0.152
20	59000000	5	0.180	0.165	0.095	0.099	0.084	0.066
21	0	33	0.081	0.101	0.068	0.084	0.013	0.017
21	1000000	48	0.086	0.067	0.013	0.033	0.073	0.033
21	2000000	61	0.093	0.098	0.102	0.110	0.009	0.012
21	3000000	60	0.084	0.116	0.165	0.214	0.081	0.097
21	4000000	53	0.179	0.227	0.198	0.178	0.019	0.050
21	5000000	60	0.171	0.185	0.146	0.145	0.025	0.040
21	6000000	78	0.085	0.073	0.153	0.168	0.068	0.095
21	7000000	68	0.163	0.175	0.131	0.137	0.032	0.038
21	8000000	79	0.180	0.143	0.172	0.167	0.008	0.024
21	9000000	75	0.125	0.075	0.138	0.180	0.013	0.106
21	10000000	65	0.222	0.183	0.240	0.218	0.017	0.035
21	11000000	64	0.200	0.194	0.225	0.226	0.025	0.032
21	12000000	69	0.241	0.225	0.222	0.218	0.019	0.007
21	13000000	60	0.214	0.240	0.145	0.149	0.069	0.091
21	14000000	67	0.162	0.136	0.143	0.161	0.018	0.026
21	15000000	79	0.183	0.173	0.199	0.194	0.016	0.020
21	16000000	62	0.223	0.203	0.189	0.184	0.034	0.018
21	17000000	74	0.178	0.176	0.213	0.210	0.035	0.034
21	18000000	79	0.197	0.183	0.203	0.210	0.006	0.027
21	19000000	83	0.215	0.202	0.156	0.187	0.059	0.015
21	20000000	63	0.185	0.193	0.076	0.136	0.108	0.057
21	21000000	65	0.143	0.150	0.180	0.209	0.037	0.059
21	22000000	62	0.204	0.194	0.119	0.134	0.085	0.061
21	23000000	69	0.128	0.128	0.215	0.231	0.087	0.103
21	24000000	79	0.175	0.178	0.213	0.202	0.038	0.025
21	25000000	69	0.150	0.165	0.175	0.133	0.024	0.032
21	26000000	68	0.191	0.215	0.203	0.182	0.012	0.033
21	27000000	64	0.158	0.165	0.184	0.213	0.025	0.049
21	28000000	69	0.171	0.180	0.190	0.211	0.020	0.031
21	29000000	66	0.151	0.138	0.211	0.227	0.060	0.089
21	30000000	58	0.138	0.183	0.199	0.205	0.061	0.022
21	31000000	81	0.159	0.159	0.174	0.195	0.015	0.036
21	32000000	78	0.155	0.147	0.165	0.224	0.010	0.077
21	33000000	76	0.204	0.168	0.206	0.220	0.002	0.052
21	34000000	81	0.141	0.169	0.170	0.191	0.029	0.022
21	35000000	71	0.153	0.144	0.174	0.171	0.021	0.027
21	36000000	57	0.119	0.144	0.176	0.192	0.057	0.049
21	37000000	62	0.124	0.121	0.171	0.176	0.047	0.056
21	38000000	80	0.189	0.205	0.176	0.181	0.014	0.024
21	39000000	68	0.190	0.182	0.127	0.139	0.063	0.043
21	40000000	67	0.151	0.159	0.199	0.181	0.048	0.021
21	41000000	72	0.166	0.173	0.195	0.194	0.030	0.021
21	42000000	82	0.224	0.207	0.187	0.182	0.036	0.024
21	43000000	70	0.158	0.152	0.148	0.161	0.010	0.008
21	44000000	69	0.119	0.164	0.158	0.172	0.039	0.008
21	45000000	68	0.186	0.195	0.170	0.180	0.016	0.015
21	46000000	71	0.187	0.198	0.176	0.172	0.012	0.027
21	47000000	82	0.168	0.183	0.129	0.137	0.039	0.047
21	48000000	81	0.174	0.185	0.149	0.161	0.025	0.024
21	49000000	88	0.145	0.162	0.120	0.128	0.025	0.034
21	50000000	64	0.169	0.200	0.118	0.122	0.051	0.078
22	0	71	0.187	0.125	0.170	0.153	0.017	0.029
22	1000000	72	0.194	0.149	0.212	0.204	0.018	0.055
22	2000000	79	0.260	0.223	0.206	0.213	0.053	0.010
22	3000000	64	0.150	0.149	0.171	0.193	0.021	0.043
22	4000000	67	0.172	0.172	0.162	0.176	0.010	0.003
22	5000000	58	0.200	0.160	0.109	0.142	0.091	0.018
22	6000000	60	0.154	0.194	0.208	0.212	0.054	0.018
22	7000000	67	0.188	0.179	0.083	0.106	0.104	0.073
22	8000000	57	0.193	0.166	0.158	0.149	0.036	0.017
22	9000000	73	0.135	0.109	0.149	0.191	0.014	0.082
22	10000000	77	0.175	0.146	0.136	0.172	0.040	0.026
22	11000000	64	0.157	0.150	0.137	0.196	0.020	0.046
22	12000000	56	0.184	0.150	0.174	0.170	0.011	0.019
22	13000000	75	0.156	0.118	0.103	0.162	0.053	0.044
22	14000000	57	0.110	0.144	0.183	0.180	0.073	0.036
22	15000000	76	0.189	0.162	0.081	0.142	0.108	0.020
22	16000000	51	0.132	0.137	0.157	0.167	0.024	0.030
22	17000000	75	0.178	0.131	0.185	0.183	0.008	0.052
22	18000000	74	0.162	0.168	0.116	0.158	0.046	0.010
22	19000000	62	0.135	0.145	0.079	0.099	0.056	0.046
22	20000000	73	0.079	0.060	0.170	0.205	0.090	0.145
22	21000000	80	0.155	0.139	0.138	0.151	0.017	0.012
22	22000000	66	0.146	0.132	0.189	0.155	0.043	0.022
22	23000000	73	0.200	0.220	0.157	0.175	0.042	0.046
22	24000000	65	0.173	0.186	0.148	0.178	0.026	0.008
22	25000000	81	0.187	0.177	0.116	0.143	0.071	0.034
22	26000000	75	0.106	0.126	0.139	0.146	0.033	0.020
22	27000000	86	0.118	0.099	0.131	0.164	0.013	0.065

22	28000000	60	0.152	0.187	0.157	0.169	0.005	0.017
22	29000000	61	0.095	0.108	0.104	0.100	0.009	0.008
22	30000000	53	0.114	0.118	0.076	0.105	0.037	0.013
22	31000000	74	0.117	0.149	0.090	0.105	0.028	0.045
22	32000000	67	0.089	0.088	0.167	0.151	0.078	0.063
22	33000000	52	0.119	0.143	0.185	0.181	0.066	0.039
22	34000000	72	0.234	0.195	0.138	0.140	0.096	0.056
22	35000000	73	0.146	0.132	0.149	0.156	0.003	0.024
22	36000000	69	0.176	0.168	0.224	0.205	0.048	0.038
22	37000000	75	0.166	0.170	0.237	0.235	0.071	0.066
22	38000000	68	0.163	0.175	0.167	0.170	0.004	0.006
22	39000000	71	0.207	0.202	0.154	0.136	0.052	0.066
22	40000000	69	0.179	0.176	0.122	0.135	0.057	0.041
22	41000000	69	0.150	0.148	0.141	0.159	0.009	0.011
22	42000000	73	0.172	0.149	0.115	0.160	0.057	0.011
22	43000000	77	0.166	0.123	0.151	0.144	0.014	0.021
22	44000000	68	0.103	0.087	0.095	0.080	0.008	0.007
22	45000000	90	0.137	0.098	0.125	0.137	0.012	0.039
22	46000000	72	0.146	0.092	0.150	0.153	0.004	0.061
22	47000000	77	0.173	0.125	0.180	0.170	0.007	0.045
22	48000000	75	0.199	0.179	0.178	0.200	0.021	0.020
22	49000000	56	0.170	0.176	0.102	0.123	0.068	0.053
22	50000000	86	0.102	0.061	0.130	0.137	0.028	0.076
22	51000000	71	0.142	0.100	0.165	0.168	0.023	0.068
22	52000000	66	0.100	0.100	0.195	0.197	0.095	0.096
22	53000000	59	0.153	0.110	0.212	0.210	0.059	0.100
22	54000000	81	0.156	0.113	0.181	0.187	0.025	0.074
22	55000000	77	0.150	0.126	0.178	0.190	0.027	0.064
22	56000000	69	0.134	0.102	0.198	0.198	0.064	0.095
22	57000000	78	0.135	0.110	0.175	0.212	0.040	0.102
22	58000000	81	0.104	0.114	0.191	0.216	0.087	0.102
22	59000000	87	0.166	0.165	0.161	0.163	0.005	0.003
22	60000000	82	0.138	0.145	0.168	0.181	0.030	0.036
22	61000000	28	0.227	0.218	0.208	0.181	0.019	0.037
22	62000000	2	0.188	0.182	0.088	0.090	0.099	0.092
23	0	53	0.067	0.059	0.100	0.097	0.032	0.038
23	1000000	50	0.105	0.089	0.078	0.065	0.027	0.024
23	2000000	59	0.179	0.182	0.156	0.111	0.023	0.071
23	3000000	60	0.192	0.179	0.175	0.194	0.017	0.015
23	4000000	59	0.148	0.172	0.119	0.149	0.029	0.023
23	5000000	64	0.145	0.132	0.165	0.184	0.020	0.052
23	6000000	78	0.176	0.143	0.155	0.160	0.021	0.017
23	7000000	83	0.164	0.115	0.182	0.152	0.018	0.037
23	8000000	70	0.217	0.186	0.129	0.134	0.088	0.052
23	9000000	81	0.153	0.133	0.123	0.128	0.030	0.005
23	10000000	65	0.165	0.130	0.126	0.136	0.039	0.006
23	11000000	84	0.132	0.132	0.214	0.214	0.082	0.081
23	12000000	81	0.150	0.135	0.162	0.165	0.012	0.029
23	13000000	77	0.170	0.168	0.138	0.157	0.031	0.010
23	14000000	85	0.161	0.108	0.174	0.168	0.013	0.060
23	15000000	72	0.140	0.117	0.122	0.198	0.018	0.081
23	16000000	81	0.202	0.169	0.111	0.127	0.091	0.042
23	17000000	71	0.119	0.118	0.123	0.139	0.004	0.020
23	18000000	83	0.134	0.115	0.184	0.214	0.050	0.100
23	19000000	77	0.170	0.127	0.158	0.183	0.012	0.056
23	20000000	64	0.215	0.189	0.140	0.156	0.074	0.034
23	21000000	76	0.175	0.137	0.140	0.136	0.035	0.000
23	22000000	64	0.175	0.169	0.161	0.167	0.014	0.002
23	23000000	74	0.212	0.169	0.129	0.117	0.083	0.052
23	24000000	78	0.155	0.114	0.089	0.102	0.066	0.012
23	25000000	70	0.099	0.085	0.181	0.226	0.082	0.141
23	26000000	67	0.109	0.118	0.200	0.213	0.091	0.095
23	27000000	70	0.173	0.159	0.199	0.177	0.026	0.018
23	28000000	71	0.145	0.156	0.202	0.211	0.057	0.056
23	29000000	64	0.157	0.148	0.102	0.124	0.055	0.023
23	30000000	80	0.168	0.171	0.159	0.177	0.009	0.005
23	31000000	81	0.143	0.148	0.139	0.112	0.004	0.036
23	32000000	64	0.117	0.140	0.219	0.175	0.102	0.035
23	33000000	63	0.183	0.202	0.153	0.161	0.030	0.041
23	34000000	66	0.152	0.138	0.100	0.123	0.051	0.015
23	35000000	75	0.152	0.136	0.131	0.150	0.021	0.014
23	36000000	81	0.144	0.156	0.094	0.123	0.049	0.033
23	37000000	79	0.115	0.149	0.132	0.153	0.017	0.004
23	38000000	66	0.098	0.121	0.136	0.156	0.037	0.035
23	39000000	71	0.114	0.138	0.139	0.164	0.025	0.026
23	40000000	75	0.082	0.121	0.124	0.118	0.042	0.003
23	41000000	61	0.114	0.124	0.136	0.171	0.023	0.047
23	42000000	69	0.157	0.177	0.118	0.147	0.039	0.030
23	43000000	71	0.135	0.133	0.097	0.158	0.038	0.025
23	44000000	77	0.163	0.151	0.186	0.206	0.023	0.056
23	45000000	71	0.171	0.184	0.178	0.176	0.007	0.008
23	46000000	78	0.175	0.147	0.129	0.161	0.046	0.014
23	47000000	71	0.169	0.179	0.146	0.154	0.024	0.024
23	48000000	80	0.105	0.120	0.116	0.125	0.011	0.006
23	49000000	80	0.142	0.135	0.129	0.135	0.012	0.000
23	50000000	71	0.125	0.111	0.113	0.117	0.012	0.006
23	51000000	54	0.122	0.140	0.039	0.093	0.083	0.047
23	52000000	20	0.160	0.174	0.000	0.000	0.160	0.174
23	54000000	1	0.000	0.000	0.052	0.059	0.052	0.059
24	0	70	0.090	0.087	0.094	0.113	0.003	0.025
24	1000000	52	0.099	0.110	0.091	0.113	0.007	0.003
24	2000000	51	0.140	0.172	0.097	0.083	0.043	0.089
24	3000000	55	0.099	0.139	0.177	0.160	0.078	0.020
24	4000000	40	0.189	0.278	0.133	0.132	0.057	0.146
24	5000000	62	0.129	0.159	0.136	0.133	0.007	0.026
24	6000000	72	0.165	0.161	0.128	0.130	0.037	0.031
24	7000000	73	0.138	0.130	0.089	0.083	0.049	0.047
24	8000000	67	0.128	0.145	0.144	0.118	0.015	0.026
24	9000000	75	0.160	0.175	0.192	0.154	0.031	0.021
24	10000000	76	0.118	0.169	0.187	0.166	0.069	0.003
24	11000000	85	0.127	0.147	0.197	0.210	0.070	0.062
24	12000000	85	0.150	0.153	0.171	0.216	0.020	0.063
24	13000000	91	0.130	0.168	0.128	0.155	0.002	0.013
24	14000000	77	0.158	0.193	0.206	0.207	0.048	0.015
24	15000000	84	0.095	0.103	0.139	0.169	0.045	0.066

24	16000000	75	0.117	0.142	0.099	0.160	0.018	0.018
24	17000000	82	0.097	0.139	0.147	0.189	0.050	0.050
24	18000000	85	0.090	0.141	0.119	0.131	0.029	0.010
24	19000000	65	0.115	0.148	0.096	0.131	0.018	0.018
24	20000000	79	0.154	0.115	0.101	0.132	0.054	0.017
24	21000000	77	0.102	0.135	0.060	0.145	0.042	0.010
24	22000000	73	0.128	0.144	0.041	0.066	0.087	0.079
24	23000000	60	0.057	0.057	0.053	0.071	0.004	0.014
24	24000000	83	0.135	0.130	0.129	0.151	0.005	0.021
24	25000000	75	0.143	0.171	0.160	0.114	0.017	0.057
24	26000000	87	0.168	0.178	0.189	0.194	0.021	0.016
24	27000000	89	0.152	0.151	0.133	0.153	0.019	0.002
24	28000000	76	0.115	0.129	0.116	0.120	0.001	0.009
24	29000000	69	0.117	0.116	0.183	0.154	0.066	0.037
24	30000000	75	0.159	0.177	0.198	0.174	0.039	0.003
24	31000000	66	0.168	0.194	0.135	0.142	0.034	0.052
24	32000000	68	0.130	0.144	0.167	0.172	0.038	0.027
24	33000000	65	0.143	0.158	0.104	0.105	0.039	0.053
24	34000000	68	0.147	0.144	0.164	0.183	0.017	0.040
24	35000000	63	0.137	0.165	0.149	0.158	0.013	0.007
24	36000000	76	0.136	0.144	0.146	0.166	0.009	0.022
24	37000000	78	0.141	0.169	0.148	0.160	0.006	0.009
24	38000000	77	0.174	0.178	0.117	0.137	0.056	0.041
24	39000000	78	0.108	0.103	0.125	0.138	0.017	0.036
24	40000000	73	0.149	0.176	0.108	0.149	0.042	0.027
24	41000000	77	0.115	0.115	0.110	0.169	0.005	0.054
24	42000000	88	0.110	0.081	0.077	0.093	0.033	0.013
24	43000000	59	0.165	0.166	0.077	0.133	0.088	0.033
24	44000000	74	0.125	0.120	0.074	0.180	0.050	0.061
24	45000000	81	0.160	0.129	0.025	0.052	0.136	0.077
24	46000000	74	0.096	0.153	0.092	0.134	0.004	0.019
24	47000000	49	0.150	0.129	0.080	0.065	0.070	0.064
25	0	40	0.070	0.075	0.087	0.112	0.017	0.036
25	1000000	46	0.085	0.096	0.169	0.223	0.084	0.127
25	2000000	74	0.230	0.156	0.188	0.222	0.042	0.066
25	3000000	67	0.209	0.241	0.198	0.204	0.011	0.037
25	4000000	72	0.189	0.234	0.184	0.166	0.006	0.068
25	5000000	67	0.181	0.232	0.174	0.156	0.007	0.076
25	6000000	52	0.128	0.164	0.133	0.168	0.004	0.004
25	7000000	70	0.153	0.154	0.101	0.127	0.053	0.027
25	8000000	70	0.127	0.142	0.170	0.195	0.043	0.054
25	9000000	61	0.140	0.141	0.152	0.215	0.012	0.074
25	10000000	83	0.181	0.141	0.137	0.158	0.044	0.017
25	11000000	78	0.172	0.143	0.139	0.180	0.034	0.037
25	12000000	69	0.106	0.111	0.160	0.192	0.054	0.081
25	13000000	62	0.150	0.140	0.153	0.158	0.003	0.019
25	14000000	77	0.153	0.144	0.244	0.198	0.091	0.054
25	15000000	62	0.136	0.144	0.184	0.180	0.048	0.036
25	16000000	87	0.158	0.097	0.164	0.134	0.006	0.037
25	17000000	64	0.128	0.092	0.099	0.220	0.029	0.128
25	18000000	69	0.163	0.138	0.150	0.183	0.012	0.046
25	19000000	53	0.172	0.142	0.168	0.175	0.005	0.033
25	20000000	58	0.218	0.192	0.213	0.216	0.005	0.025
25	21000000	68	0.188	0.185	0.169	0.188	0.019	0.003
25	22000000	66	0.169	0.166	0.132	0.136	0.038	0.030
25	23000000	79	0.165	0.143	0.139	0.166	0.026	0.023
25	24000000	80	0.150	0.133	0.214	0.215	0.064	0.082
25	25000000	63	0.186	0.182	0.238	0.232	0.052	0.050
25	26000000	67	0.183	0.199	0.193	0.192	0.010	0.007
25	27000000	80	0.189	0.169	0.200	0.189	0.011	0.020
25	28000000	83	0.178	0.149	0.092	0.135	0.086	0.014
25	29000000	82	0.142	0.134	0.198	0.202	0.056	0.067
25	30000000	84	0.149	0.178	0.193	0.165	0.044	0.013
25	31000000	78	0.160	0.145	0.074	0.092	0.085	0.053
25	32000000	78	0.124	0.141	0.076	0.089	0.049	0.051
25	33000000	75	0.113	0.111	0.168	0.173	0.054	0.062
25	34000000	77	0.156	0.146	0.150	0.170	0.007	0.024
25	35000000	75	0.199	0.187	0.131	0.139	0.069	0.048
25	36000000	81	0.151	0.147	0.188	0.182	0.037	0.036
25	37000000	73	0.192	0.204	0.159	0.149	0.033	0.055
25	38000000	81	0.147	0.153	0.127	0.118	0.020	0.035
25	39000000	77	0.130	0.125	0.138	0.131	0.008	0.006
25	40000000	74	0.139	0.151	0.135	0.147	0.004	0.004
25	41000000	68	0.139	0.157	0.132	0.140	0.007	0.017
25	42000000	81	0.173	0.207	0.148	0.161	0.026	0.046
25	43000000	74	0.182	0.209	0.135	0.124	0.047	0.085
25	44000000	83	0.190	0.176	0.142	0.153	0.048	0.023
25	45000000	86	0.154	0.152	0.134	0.168	0.020	0.017
25	46000000	87	0.191	0.177	0.156	0.190	0.035	0.013
25	47000000	83	0.147	0.171	0.152	0.181	0.006	0.011
25	48000000	81	0.191	0.188	0.146	0.161	0.045	0.027
25	49000000	80	0.137	0.139	0.094	0.147	0.043	0.008
25	50000000	45	0.128	0.134	0.097	0.091	0.031	0.043
25	51000000	46	0.104	0.134	0.073	0.119	0.031	0.015
25	53000000	4	0.170	0.183	0.138	0.200	0.032	0.017
26	0	83	0.177	0.132	0.170	0.223	0.007	0.091
26	1000000	77	0.196	0.159	0.148	0.159	0.048	0.001
26	2000000	70	0.151	0.161	0.195	0.218	0.045	0.057
26	3000000	69	0.168	0.198	0.194	0.218	0.026	0.020
26	4000000	73	0.197	0.193	0.167	0.188	0.030	0.006
26	5000000	68	0.179	0.151	0.152	0.196	0.027	0.045
26	6000000	76	0.194	0.199	0.108	0.119	0.086	0.080
26	7000000	73	0.165	0.163	0.108	0.087	0.057	0.076
26	8000000	58	0.139	0.166	0.127	0.160	0.011	0.007
26	9000000	55	0.132	0.124	0.168	0.173	0.036	0.049
26	10000000	92	0.177	0.192	0.185	0.220	0.008	0.028
26	11000000	77	0.235	0.173	0.150	0.161	0.084	0.013
26	12000000	72	0.173	0.169	0.166	0.192	0.008	0.022
26	13000000	81	0.179	0.176	0.153	0.175	0.026	0.002
26	14000000	82	0.138	0.135	0.224	0.221	0.087	0.086
26	15000000	81	0.171	0.143	0.185	0.187	0.013	0.044
26	16000000	61	0.195	0.207	0.207	0.247	0.012	0.040
26	17000000	73	0.210	0.210	0.096	0.133	0.114	0.078
26	18000000	74	0.130	0.142	0.151	0.168	0.022	0.026
26	19000000	82	0.186	0.196	0.158	0.175	0.028	0.021

26	20000000	80	0.170	0.169	0.089	0.095	0.081	0.074
26	21000000	58	0.085	0.098	0.156	0.150	0.071	0.053
26	22000000	64	0.169	0.150	0.130	0.135	0.038	0.015
26	23000000	60	0.183	0.152	0.143	0.158	0.040	0.006
26	24000000	67	0.165	0.169	0.153	0.168	0.012	0.001
26	25000000	13	0.080	0.060	0.072	0.101	0.009	0.041
26	26000000	20	0.002	0.030	0.121	0.107	0.119	0.077
26	27000000	47	0.126	0.123	0.123	0.163	0.002	0.040
26	28000000	70	0.152	0.159	0.131	0.156	0.020	0.003
26	29000000	72	0.126	0.102	0.149	0.161	0.023	0.058
26	30000000	59	0.145	0.161	0.168	0.162	0.022	0.002
26	31000000	66	0.158	0.144	0.205	0.208	0.047	0.064
26	32000000	77	0.173	0.185	0.175	0.198	0.002	0.013
26	33000000	80	0.138	0.144	0.176	0.197	0.039	0.052
26	34000000	68	0.177	0.201	0.188	0.194	0.011	0.008
26	35000000	75	0.146	0.181	0.217	0.225	0.072	0.045
26	36000000	92	0.205	0.221	0.163	0.167	0.042	0.054
26	37000000	81	0.144	0.126	0.137	0.183	0.007	0.057
26	38000000	80	0.160	0.180	0.159	0.165	0.002	0.015
27	0	58	0.112	0.111	0.195	0.219	0.083	0.109
27	1000000	71	0.170	0.168	0.196	0.196	0.026	0.028
27	2000000	89	0.198	0.180	0.184	0.185	0.015	0.006
27	3000000	67	0.186	0.164	0.153	0.169	0.033	0.005
27	4000000	71	0.148	0.143	0.152	0.165	0.004	0.021
27	5000000	62	0.194	0.165	0.159	0.173	0.035	0.008
27	6000000	63	0.174	0.148	0.093	0.097	0.081	0.051
27	7000000	71	0.154	0.133	0.183	0.174	0.029	0.041
27	8000000	69	0.125	0.104	0.148	0.178	0.023	0.074
27	9000000	71	0.096	0.108	0.179	0.166	0.083	0.058
27	10000000	75	0.117	0.120	0.185	0.197	0.068	0.077
27	11000000	61	0.167	0.142	0.195	0.202	0.028	0.060
27	12000000	79	0.157	0.093	0.164	0.162	0.007	0.069
27	13000000	72	0.171	0.139	0.190	0.180	0.019	0.041
27	14000000	70	0.140	0.154	0.186	0.180	0.046	0.026
27	15000000	63	0.180	0.177	0.194	0.190	0.014	0.012
27	16000000	73	0.171	0.186	0.121	0.146	0.050	0.040
27	17000000	67	0.136	0.154	0.186	0.173	0.051	0.019
27	18000000	77	0.156	0.174	0.162	0.159	0.006	0.015
27	19000000	66	0.171	0.167	0.169	0.182	0.002	0.015
27	20000000	64	0.109	0.112	0.200	0.195	0.091	0.083
27	21000000	74	0.144	0.157	0.171	0.193	0.026	0.035
27	22000000	67	0.139	0.156	0.194	0.188	0.055	0.032
27	23000000	71	0.150	0.151	0.213	0.197	0.063	0.047
27	24000000	78	0.199	0.186	0.204	0.197	0.005	0.011
27	25000000	59	0.189	0.181	0.154	0.179	0.035	0.002
27	26000000	69	0.193	0.182	0.186	0.178	0.007	0.004
27	27000000	64	0.147	0.167	0.161	0.149	0.015	0.019
27	28000000	69	0.134	0.123	0.119	0.129	0.014	0.006
27	29000000	70	0.075	0.095	0.202	0.172	0.127	0.077
27	30000000	75	0.155	0.155	0.163	0.182	0.008	0.027
27	31000000	76	0.142	0.138	0.159	0.170	0.017	0.032
27	32000000	71	0.187	0.169	0.188	0.177	0.001	0.008
27	33000000	74	0.152	0.151	0.165	0.159	0.013	0.008
27	34000000	65	0.156	0.156	0.177	0.201	0.021	0.045
27	35000000	53	0.143	0.181	0.195	0.185	0.051	0.004
27	36000000	62	0.182	0.181	0.153	0.151	0.029	0.031
27	37000000	57	0.128	0.167	0.202	0.198	0.074	0.032
27	38000000	62	0.182	0.192	0.226	0.210	0.044	0.018
27	39000000	86	0.212	0.199	0.174	0.183	0.038	0.017
27	40000000	72	0.157	0.156	0.149	0.189	0.009	0.034
27	41000000	79	0.188	0.192	0.160	0.166	0.027	0.026
27	42000000	82	0.151	0.150	0.058	0.093	0.093	0.057
27	43000000	60	0.116	0.116	0.137	0.226	0.022	0.111
27	44000000	90	0.247	0.248	0.120	0.148	0.127	0.101
27	45000000	40	0.101	0.154	0.136	0.165	0.035	0.011
28	0	58	0.159	0.126	0.181	0.187	0.022	0.061
28	1000000	56	0.145	0.154	0.133	0.136	0.013	0.018
28	2000000	57	0.145	0.147	0.113	0.135	0.032	0.013
28	3000000	57	0.184	0.185	0.130	0.114	0.054	0.071
28	4000000	58	0.130	0.121	0.121	0.178	0.009	0.058
28	5000000	55	0.146	0.133	0.136	0.174	0.010	0.041
28	6000000	40	0.062	0.114	0.094	0.111	0.032	0.002
28	7000000	60	0.120	0.100	0.076	0.114	0.044	0.014
28	8000000	70	0.143	0.113	0.047	0.111	0.096	0.002
28	9000000	49	0.073	0.125	0.056	0.105	0.017	0.021
28	10000000	62	0.145	0.160	0.099	0.133	0.046	0.027
28	11000000	76	0.177	0.151	0.199	0.191	0.022	0.040
28	12000000	66	0.138	0.117	0.118	0.178	0.020	0.060
28	13000000	65	0.150	0.130	0.101	0.142	0.049	0.012
28	14000000	65	0.115	0.112	0.122	0.184	0.006	0.072
28	15000000	79	0.195	0.144	0.109	0.147	0.087	0.002
28	16000000	73	0.219	0.177	0.145	0.162	0.075	0.015
28	17000000	74	0.154	0.146	0.152	0.202	0.001	0.056
28	18000000	64	0.187	0.167	0.169	0.183	0.019	0.016
28	19000000	79	0.198	0.149	0.144	0.183	0.055	0.034
28	20000000	80	0.179	0.165	0.183	0.219	0.004	0.054
28	21000000	79	0.166	0.175	0.161	0.151	0.005	0.024
28	22000000	71	0.173	0.142	0.209	0.222	0.035	0.080
28	23000000	79	0.218	0.172	0.165	0.171	0.054	0.001
28	24000000	74	0.132	0.141	0.089	0.100	0.043	0.041
28	25000000	79	0.180	0.164	0.140	0.138	0.039	0.026
28	26000000	68	0.144	0.159	0.074	0.095	0.071	0.064
28	27000000	72	0.097	0.108	0.122	0.133	0.025	0.025
28	28000000	73	0.177	0.163	0.168	0.170	0.009	0.007
28	29000000	70	0.192	0.162	0.162	0.181	0.030	0.019
28	30000000	81	0.225	0.217	0.183	0.201	0.042	0.017
28	31000000	85	0.133	0.109	0.156	0.193	0.023	0.084
28	32000000	74	0.176	0.187	0.140	0.170	0.037	0.017
28	33000000	81	0.149	0.163	0.161	0.176	0.012	0.013
28	34000000	77	0.159	0.171	0.193	0.214	0.034	0.043
28	35000000	92	0.164	0.170	0.195	0.211	0.032	0.040
28	36000000	88	0.181	0.191	0.179	0.193	0.002	0.002
28	37000000	85	0.165	0.148	0.196	0.223	0.031	0.075
28	38000000	84	0.230	0.212	0.168	0.187	0.062	0.025
28	39000000	81	0.181	0.172	0.181	0.187	0.000	0.015

28	40000000	70	0.135	0.136	0.112	0.184	0.022	0.048
28	41000000	7	0.107	0.094	0.129	0.152	0.022	0.057
29	0	45	0.136	0.158	0.066	0.101	0.070	0.058
29	1000000	76	0.128	0.097	0.156	0.185	0.028	0.088
29	2000000	69	0.184	0.175	0.141	0.171	0.043	0.004
29	3000000	63	0.130	0.142	0.156	0.186	0.026	0.044
29	4000000	61	0.183	0.183	0.138	0.170	0.045	0.012
29	5000000	77	0.135	0.131	0.130	0.187	0.004	0.056
29	6000000	56	0.148	0.181	0.153	0.172	0.005	0.009
29	7000000	74	0.152	0.152	0.074	0.131	0.078	0.021
29	8000000	71	0.156	0.146	0.110	0.135	0.046	0.011
29	9000000	70	0.133	0.141	0.141	0.155	0.009	0.014
29	10000000	77	0.178	0.167	0.091	0.137	0.087	0.030
29	11000000	77	0.172	0.157	0.115	0.141	0.057	0.016
29	12000000	71	0.172	0.145	0.145	0.177	0.026	0.032
29	13000000	76	0.190	0.145	0.139	0.142	0.051	0.002
29	14000000	77	0.177	0.129	0.100	0.098	0.077	0.032
29	15000000	56	0.109	0.083	0.136	0.163	0.026	0.080
29	16000000	57	0.109	0.092	0.184	0.188	0.075	0.096
29	17000000	73	0.171	0.129	0.171	0.178	0.000	0.048
29	18000000	70	0.162	0.150	0.094	0.108	0.069	0.042
29	19000000	72	0.141	0.127	0.191	0.206	0.050	0.079
29	20000000	71	0.120	0.110	0.179	0.178	0.059	0.068
29	21000000	79	0.128	0.110	0.093	0.155	0.035	0.045
29	22000000	68	0.113	0.076	0.175	0.236	0.062	0.159
29	23000000	74	0.168	0.095	0.150	0.166	0.018	0.070
29	24000000	73	0.148	0.101	0.091	0.144	0.057	0.043
29	25000000	63	0.175	0.139	0.132	0.160	0.043	0.021
29	26000000	64	0.116	0.112	0.094	0.132	0.022	0.020
29	27000000	69	0.130	0.146	0.183	0.158	0.053	0.012
29	28000000	66	0.197	0.207	0.088	0.098	0.109	0.109
29	29000000	68	0.100	0.118	0.130	0.152	0.030	0.034
29	30000000	68	0.128	0.139	0.226	0.249	0.098	0.110
29	31000000	77	0.172	0.150	0.156	0.139	0.016	0.011
29	32000000	76	0.215	0.246	0.170	0.196	0.045	0.050
29	33000000	78	0.211	0.224	0.211	0.211	0.000	0.013
29	34000000	70	0.202	0.204	0.169	0.184	0.033	0.020
29	35000000	68	0.133	0.154	0.134	0.173	0.001	0.020
29	36000000	57	0.159	0.160	0.158	0.177	0.001	0.017
29	37000000	51	0.179	0.166	0.149	0.144	0.030	0.022
29	38000000	56	0.143	0.146	0.141	0.162	0.002	0.016
29	39000000	53	0.149	0.150	0.203	0.211	0.054	0.061
29	40000000	45	0.171	0.182	0.153	0.152	0.019	0.031
29	41000000	52	0.181	0.176	0.117	0.121	0.064	0.055
30	0	42	0.130	0.106	0.011	0.014	0.119	0.092
30	1000000	79	0.143	0.085	0.096	0.107	0.048	0.022
30	2000000	68	0.140	0.114	0.122	0.182	0.018	0.068
30	3000000	59	0.143	0.178	0.144	0.197	0.001	0.018
30	4000000	58	0.162	0.202	0.046	0.092	0.116	0.110
30	5000000	45	0.050	0.022	0.036	0.048	0.014	0.025
30	6000000	41	0.052	0.053	0.134	0.162	0.082	0.109
30	7000000	63	0.163	0.141	0.181	0.197	0.018	0.056
30	8000000	74	0.120	0.156	0.213	0.193	0.094	0.037
30	9000000	61	0.143	0.132	0.212	0.207	0.069	0.075
30	10000000	65	0.236	0.210	0.162	0.186	0.074	0.024
30	11000000	64	0.146	0.113	0.120	0.135	0.026	0.022
30	12000000	56	0.104	0.119	0.139	0.145	0.034	0.026
30	13000000	57	0.166	0.120	0.095	0.135	0.070	0.015
30	14000000	68	0.107	0.111	0.135	0.151	0.028	0.040
30	15000000	70	0.144	0.110	0.155	0.166	0.011	0.056
30	16000000	60	0.122	0.149	0.160	0.137	0.038	0.012
30	17000000	71	0.168	0.134	0.162	0.160	0.006	0.026
30	18000000	46	0.064	0.126	0.092	0.103	0.029	0.024
30	19000000	50	0.207	0.274	0.158	0.158	0.049	0.115
30	20000000	63	0.197	0.232	0.138	0.168	0.058	0.064
30	21000000	73	0.063	0.035	0.180	0.157	0.117	0.122
30	22000000	90	0.051	0.030	0.189	0.178	0.138	0.148
30	23000000	87	0.029	0.016	0.193	0.210	0.164	0.194
30	24000000	93	0.066	0.038	0.231	0.204	0.165	0.166
30	25000000	86	0.115	0.089	0.187	0.173	0.072	0.084
30	26000000	86	0.163	0.150	0.180	0.195	0.017	0.045
30	27000000	78	0.146	0.141	0.151	0.146	0.005	0.005
30	28000000	72	0.124	0.114	0.168	0.195	0.044	0.081
30	29000000	73	0.125	0.112	0.183	0.178	0.058	0.066
30	30000000	65	0.141	0.148	0.199	0.232	0.058	0.084
30	31000000	71	0.139	0.156	0.194	0.213	0.055	0.058
30	32000000	76	0.165	0.190	0.171	0.181	0.006	0.010
30	33000000	67	0.146	0.153	0.174	0.182	0.028	0.029
30	34000000	68	0.160	0.126	0.184	0.207	0.024	0.082
30	35000000	83	0.157	0.117	0.167	0.185	0.011	0.068
30	36000000	64	0.152	0.141	0.198	0.225	0.046	0.084
30	37000000	81	0.182	0.157	0.140	0.159	0.042	0.002
30	38000000	78	0.122	0.091	0.164	0.123	0.042	0.032
30	39000000	80	0.143	0.098	0.134	0.176	0.009	0.078
30	40000000	19	0.142	0.140	0.121	0.137	0.021	0.003
31	0	70	0.151	0.154	0.162	0.189	0.011	0.035
31	1000000	57	0.235	0.244	0.156	0.132	0.079	0.112
31	2000000	76	0.128	0.102	0.111	0.143	0.017	0.041
31	3000000	68	0.149	0.164	0.164	0.096	0.015	0.068
31	4000000	65	0.162	0.187	0.206	0.172	0.043	0.015
31	5000000	76	0.176	0.164	0.117	0.156	0.059	0.008
31	6000000	76	0.186	0.170	0.154	0.168	0.032	0.002
31	7000000	72	0.213	0.210	0.140	0.152	0.073	0.059
31	8000000	69	0.183	0.183	0.135	0.131	0.048	0.052
31	9000000	64	0.162	0.201	0.134	0.151	0.028	0.050
31	10000000	57	0.179	0.199	0.108	0.125	0.071	0.074
31	11000000	63	0.115	0.112	0.144	0.158	0.029	0.046
31	12000000	54	0.171	0.203	0.138	0.144	0.033	0.059
31	13000000	66	0.170	0.173	0.124	0.151	0.045	0.022
31	14000000	71	0.168	0.175	0.085	0.127	0.083	0.048
31	15000000	65	0.169	0.202	0.153	0.170	0.015	0.033
31	16000000	69	0.149	0.122	0.145	0.164	0.004	0.042
31	17000000	65	0.220	0.203	0.169	0.180	0.051	0.023
31	18000000	69	0.230	0.217	0.150	0.164	0.080	0.053
31	19000000	71	0.222	0.211	0.142	0.152	0.081	0.059

31	20000000	64	0.174	0.185	0.134	0.161	0.040	0.024
31	21000000	68	0.169	0.171	0.132	0.153	0.038	0.018
31	22000000	65	0.202	0.225	0.110	0.138	0.093	0.086
31	23000000	66	0.164	0.165	0.086	0.146	0.078	0.019
31	24000000	68	0.198	0.195	0.116	0.122	0.082	0.073
31	25000000	60	0.171	0.169	0.068	0.063	0.103	0.106
31	26000000	65	0.078	0.089	0.181	0.209	0.103	0.120
31	27000000	45	0.187	0.169	0.198	0.107	0.011	0.061
31	28000000	15	0.125	0.130	0.104	0.141	0.021	0.011
31	29000000	52	0.117	0.131	0.179	0.175	0.062	0.043
31	30000000	81	0.148	0.131	0.164	0.196	0.016	0.065
31	31000000	86	0.154	0.146	0.173	0.195	0.018	0.049
31	32000000	71	0.152	0.159	0.202	0.198	0.050	0.040
31	33000000	63	0.140	0.146	0.203	0.225	0.062	0.079
31	34000000	85	0.183	0.189	0.165	0.207	0.018	0.018
31	35000000	79	0.125	0.147	0.211	0.207	0.086	0.060
31	36000000	93	0.201	0.184	0.164	0.186	0.036	0.001
31	37000000	74	0.171	0.164	0.153	0.169	0.018	0.005
31	38000000	68	0.193	0.198	0.153	0.163	0.040	0.035
31	39000000	54	0.134	0.138	0.103	0.112	0.031	0.026
32	0	50	0.141	0.156	0.101	0.140	0.040	0.016
32	1000000	67	0.115	0.125	0.173	0.159	0.058	0.034
32	2000000	63	0.116	0.114	0.150	0.182	0.034	0.068
32	3000000	65	0.132	0.125	0.121	0.190	0.011	0.065
32	4000000	76	0.155	0.147	0.142	0.167	0.014	0.020
32	5000000	65	0.155	0.172	0.148	0.161	0.007	0.011
32	6000000	71	0.165	0.180	0.174	0.190	0.009	0.010
32	7000000	66	0.129	0.133	0.146	0.158	0.018	0.025
32	8000000	79	0.119	0.102	0.125	0.149	0.006	0.046
32	9000000	80	0.131	0.125	0.180	0.179	0.048	0.054
32	10000000	72	0.137	0.124	0.134	0.170	0.004	0.045
32	11000000	69	0.131	0.098	0.161	0.201	0.030	0.103
32	12000000	71	0.127	0.086	0.144	0.175	0.016	0.089
32	13000000	81	0.159	0.129	0.128	0.175	0.031	0.046
32	14000000	73	0.165	0.150	0.190	0.217	0.025	0.067
32	15000000	59	0.204	0.218	0.153	0.170	0.051	0.049
32	16000000	70	0.156	0.148	0.190	0.204	0.035	0.057
32	17000000	80	0.150	0.119	0.157	0.203	0.008	0.083
32	18000000	68	0.185	0.149	0.177	0.202	0.008	0.053
32	19000000	72	0.164	0.157	0.129	0.166	0.035	0.010
32	20000000	82	0.107	0.091	0.117	0.163	0.010	0.072
32	21000000	84	0.111	0.114	0.169	0.226	0.058	0.112
32	22000000	75	0.162	0.178	0.177	0.192	0.015	0.014
32	23000000	63	0.210	0.218	0.158	0.158	0.052	0.060
32	24000000	62	0.153	0.172	0.197	0.224	0.045	0.052
32	25000000	61	0.201	0.195	0.157	0.176	0.044	0.020
32	26000000	67	0.162	0.175	0.118	0.142	0.044	0.032
32	27000000	58	0.136	0.129	0.171	0.187	0.034	0.058
32	28000000	70	0.152	0.133	0.122	0.156	0.030	0.023
32	29000000	68	0.143	0.142	0.193	0.189	0.050	0.047
32	30000000	67	0.166	0.154	0.113	0.157	0.053	0.003
32	31000000	66	0.102	0.120	0.194	0.197	0.091	0.077
32	32000000	74	0.100	0.115	0.140	0.188	0.040	0.073
32	33000000	66	0.165	0.177	0.180	0.195	0.015	0.018
32	34000000	77	0.136	0.177	0.181	0.188	0.045	0.011
32	35000000	65	0.085	0.117	0.162	0.174	0.077	0.057
32	36000000	62	0.091	0.133	0.155	0.202	0.065	0.069
32	37000000	67	0.052	0.068	0.184	0.203	0.133	0.136
32	38000000	47	0.115	0.153	0.130	0.113	0.014	0.040
33	0	53	0.204	0.216	0.113	0.116	0.091	0.100
33	1000000	71	0.168	0.167	0.204	0.181	0.036	0.014
33	2000000	78	0.149	0.135	0.196	0.216	0.046	0.081
33	3000000	69	0.121	0.130	0.132	0.105	0.011	0.025
33	4000000	64	0.167	0.159	0.147	0.153	0.020	0.005
33	5000000	71	0.137	0.137	0.189	0.200	0.052	0.063
33	6000000	80	0.133	0.129	0.176	0.182	0.043	0.053
33	7000000	73	0.119	0.141	0.194	0.162	0.074	0.021
33	8000000	78	0.149	0.138	0.186	0.180	0.037	0.042
33	9000000	72	0.177	0.131	0.173	0.183	0.004	0.052
33	10000000	80	0.198	0.180	0.207	0.196	0.009	0.016
33	11000000	73	0.119	0.149	0.162	0.163	0.043	0.015
33	12000000	69	0.144	0.144	0.126	0.130	0.018	0.014
33	13000000	65	0.134	0.168	0.189	0.179	0.055	0.011
33	14000000	62	0.162	0.183	0.233	0.204	0.071	0.021
33	15000000	76	0.190	0.200	0.169	0.170	0.021	0.031
33	16000000	85	0.185	0.170	0.199	0.203	0.014	0.033
33	17000000	80	0.181	0.178	0.158	0.137	0.022	0.042
33	18000000	67	0.156	0.156	0.161	0.193	0.005	0.037
33	19000000	71	0.135	0.130	0.196	0.203	0.060	0.073
33	20000000	70	0.224	0.210	0.168	0.201	0.056	0.009
33	21000000	71	0.161	0.157	0.198	0.205	0.037	0.047
33	22000000	70	0.173	0.182	0.109	0.119	0.065	0.063
33	23000000	59	0.128	0.121	0.124	0.128	0.004	0.007
33	24000000	65	0.192	0.177	0.170	0.189	0.021	0.012
33	25000000	78	0.200	0.201	0.125	0.166	0.075	0.034
33	26000000	75	0.171	0.171	0.172	0.201	0.001	0.029
33	27000000	78	0.201	0.195	0.194	0.187	0.007	0.008
33	28000000	66	0.171	0.155	0.206	0.201	0.035	0.047
33	29000000	69	0.193	0.227	0.160	0.169	0.033	0.058
33	30000000	79	0.134	0.151	0.203	0.191	0.069	0.039
33	31000000	27	0.144	0.115	0.000	0.000	0.144	0.115
33	33000000	1	0.000	0.000	0.124	0.089	0.124	0.089
34	0	72	0.125	0.120	0.110	0.084	0.015	0.035
34	1000000	82	0.064	0.037	0.284	0.201	0.220	0.164
34	2000000	83	0.103	0.120	0.258	0.252	0.155	0.132
34	3000000	81	0.190	0.232	0.197	0.197	0.007	0.035
34	4000000	84	0.162	0.154	0.205	0.196	0.043	0.042
34	5000000	78	0.159	0.170	0.160	0.153	0.001	0.017
34	6000000	60	0.146	0.146	0.184	0.181	0.039	0.035
34	7000000	66	0.206	0.195	0.208	0.210	0.002	0.015
34	8000000	74	0.148	0.130	0.185	0.185	0.037	0.055
34	9000000	84	0.186	0.194	0.178	0.178	0.007	0.016
34	10000000	74	0.140	0.158	0.186	0.201	0.046	0.043
34	11000000	65	0.186	0.201	0.146	0.172	0.040	0.029
34	12000000	53	0.172	0.186	0.209	0.232	0.037	0.046

34	13000000	64	0.188	0.209	0.139	0.108	0.049	0.101
34	14000000	55	0.107	0.100	0.156	0.161	0.048	0.061
34	15000000	68	0.113	0.134	0.075	0.091	0.037	0.043
34	16000000	66	0.118	0.141	0.117	0.147	0.001	0.007
34	17000000	68	0.132	0.179	0.136	0.210	0.004	0.032
34	18000000	71	0.183	0.191	0.128	0.188	0.055	0.004
34	19000000	77	0.159	0.150	0.141	0.144	0.018	0.006
34	20000000	84	0.158	0.112	0.169	0.153	0.011	0.041
34	21000000	71	0.172	0.148	0.202	0.220	0.030	0.071
34	22000000	64	0.146	0.133	0.170	0.194	0.023	0.060
34	23000000	65	0.129	0.106	0.171	0.175	0.043	0.069
34	24000000	75	0.146	0.109	0.194	0.193	0.048	0.083
34	25000000	71	0.164	0.135	0.136	0.130	0.028	0.005
34	26000000	72	0.120	0.112	0.158	0.136	0.038	0.024
34	27000000	75	0.143	0.161	0.138	0.157	0.004	0.003
34	28000000	73	0.183	0.190	0.154	0.142	0.029	0.048
34	29000000	77	0.181	0.184	0.133	0.140	0.049	0.045
34	30000000	75	0.171	0.161	0.156	0.157	0.015	0.003
34	31000000	71	0.183	0.183	0.133	0.127	0.050	0.056
34	32000000	71	0.161	0.147	0.134	0.182	0.027	0.034
34	33000000	73	0.185	0.170	0.137	0.183	0.048	0.014
34	34000000	65	0.189	0.194	0.123	0.200	0.066	0.005
34	35000000	82	0.180	0.186	0.163	0.213	0.017	0.027
34	36000000	79	0.170	0.176	0.166	0.212	0.004	0.036
34	37000000	76	0.163	0.174	0.165	0.178	0.002	0.004
34	38000000	77	0.207	0.163	0.110	0.153	0.098	0.010
34	39000000	64	0.176	0.166	0.194	0.206	0.019	0.040
34	40000000	75	0.185	0.150	0.187	0.202	0.001	0.051
34	41000000	78	0.195	0.185	0.188	0.201	0.008	0.016
34	42000000	12	0.090	0.066	0.042	0.038	0.049	0.029
34	43000000	1	0.042	0.023	0.055	0.063	0.013	0.040
35	0	55	0.086	0.099	0.140	0.181	0.054	0.082
35	1000000	71	0.158	0.135	0.129	0.146	0.029	0.011
35	2000000	71	0.125	0.142	0.138	0.187	0.013	0.045
35	3000000	77	0.136	0.156	0.093	0.196	0.043	0.039
35	4000000	73	0.167	0.198	0.104	0.159	0.063	0.040
35	5000000	83	0.165	0.161	0.188	0.206	0.023	0.045
35	6000000	89	0.181	0.214	0.140	0.152	0.041	0.061
35	7000000	80	0.173	0.186	0.162	0.195	0.011	0.010
35	8000000	83	0.152	0.173	0.159	0.161	0.007	0.012
35	9000000	74	0.138	0.129	0.166	0.211	0.028	0.082
35	10000000	80	0.189	0.188	0.133	0.210	0.055	0.022
35	11000000	86	0.181	0.179	0.153	0.164	0.028	0.014
35	12000000	77	0.174	0.163	0.158	0.205	0.016	0.041
35	13000000	68	0.193	0.207	0.168	0.206	0.026	0.001
35	14000000	68	0.164	0.176	0.148	0.198	0.016	0.022
35	15000000	80	0.196	0.177	0.153	0.186	0.043	0.008
35	16000000	72	0.137	0.147	0.181	0.196	0.044	0.049
35	17000000	81	0.176	0.179	0.121	0.140	0.056	0.040
35	18000000	65	0.148	0.144	0.222	0.242	0.073	0.098
35	19000000	75	0.102	0.125	0.174	0.192	0.072	0.068
35	20000000	84	0.210	0.219	0.182	0.184	0.028	0.034
35	21000000	82	0.154	0.148	0.176	0.198	0.022	0.051
35	22000000	76	0.188	0.158	0.179	0.198	0.010	0.040
35	23000000	70	0.125	0.109	0.158	0.164	0.033	0.054
35	24000000	70	0.160	0.188	0.173	0.182	0.014	0.006
35	25000000	61	0.192	0.190	0.184	0.195	0.008	0.005
35	26000000	24	0.212	0.213	0.142	0.119	0.070	0.094
36	0	57	0.163	0.132	0.100	0.097	0.063	0.035
36	1000000	68	0.111	0.119	0.175	0.194	0.064	0.074
36	2000000	57	0.100	0.102	0.143	0.153	0.043	0.051
36	3000000	73	0.158	0.156	0.184	0.176	0.026	0.020
36	4000000	79	0.158	0.169	0.206	0.197	0.047	0.028
36	5000000	64	0.152	0.153	0.151	0.177	0.001	0.024
36	6000000	65	0.184	0.198	0.149	0.168	0.035	0.030
36	7000000	73	0.109	0.143	0.117	0.148	0.009	0.005
36	8000000	64	0.152	0.197	0.160	0.183	0.008	0.013
36	9000000	80	0.166	0.176	0.155	0.149	0.011	0.028
36	10000000	68	0.135	0.142	0.148	0.128	0.013	0.015
36	11000000	62	0.132	0.165	0.162	0.173	0.030	0.008
36	12000000	68	0.118	0.114	0.153	0.179	0.035	0.065
36	13000000	50	0.144	0.163	0.122	0.156	0.022	0.006
36	14000000	37	0.179	0.156	0.130	0.132	0.049	0.024
36	15000000	35	0.205	0.203	0.122	0.148	0.083	0.055
36	16000000	76	0.172	0.153	0.168	0.181	0.004	0.027
36	17000000	82	0.210	0.223	0.180	0.187	0.030	0.036
36	18000000	70	0.206	0.210	0.234	0.230	0.028	0.020
36	19000000	84	0.181	0.193	0.168	0.168	0.013	0.025
36	20000000	89	0.178	0.155	0.183	0.180	0.005	0.025
36	21000000	75	0.156	0.155	0.186	0.190	0.030	0.036
36	22000000	76	0.132	0.154	0.200	0.206	0.068	0.052
36	23000000	74	0.174	0.171	0.150	0.177	0.023	0.006
36	24000000	75	0.141	0.134	0.130	0.161	0.011	0.026
36	25000000	76	0.197	0.140	0.194	0.195	0.002	0.055
36	26000000	81	0.162	0.153	0.140	0.176	0.022	0.023
36	27000000	76	0.166	0.174	0.156	0.170	0.009	0.004
36	28000000	75	0.160	0.165	0.187	0.212	0.026	0.047
36	29000000	77	0.141	0.144	0.130	0.188	0.011	0.044
36	30000000	54	0.167	0.187	0.163	0.195	0.004	0.009
37	0	61	0.102	0.165	0.177	0.185	0.074	0.021
37	1000000	63	0.172	0.229	0.222	0.252	0.050	0.022
37	2000000	67	0.182	0.178	0.228	0.249	0.046	0.071
37	3000000	55	0.209	0.237	0.080	0.084	0.129	0.153
37	4000000	68	0.108	0.110	0.155	0.153	0.047	0.043
37	5000000	76	0.164	0.165	0.179	0.189	0.015	0.024
37	6000000	80	0.102	0.115	0.073	0.080	0.029	0.035
37	7000000	67	0.063	0.063	0.138	0.149	0.076	0.087
37	8000000	85	0.169	0.154	0.192	0.196	0.023	0.042
37	9000000	77	0.141	0.167	0.116	0.098	0.024	0.069
37	10000000	62	0.111	0.092	0.086	0.079	0.026	0.013
37	11000000	56	0.127	0.111	0.128	0.134	0.000	0.024
37	12000000	67	0.137	0.122	0.159	0.156	0.022	0.034
37	13000000	74	0.125	0.118	0.201	0.205	0.077	0.087
37	14000000	70	0.164	0.195	0.149	0.144	0.016	0.050
37	15000000	73	0.155	0.147	0.105	0.118	0.050	0.029

37	16000000	49	0.110	0.150	0.134	0.161	0.024	0.010
37	17000000	72	0.163	0.142	0.135	0.153	0.028	0.011
37	18000000	66	0.174	0.180	0.118	0.150	0.056	0.030
37	19000000	75	0.181	0.160	0.168	0.196	0.013	0.037
37	20000000	72	0.155	0.123	0.197	0.201	0.042	0.078
37	21000000	73	0.185	0.178	0.201	0.201	0.016	0.024
37	22000000	69	0.178	0.176	0.153	0.159	0.026	0.017
37	23000000	74	0.174	0.179	0.160	0.182	0.014	0.004
37	24000000	67	0.217	0.195	0.145	0.188	0.071	0.008
37	25000000	72	0.146	0.143	0.145	0.187	0.001	0.044
37	26000000	78	0.174	0.179	0.167	0.183	0.006	0.004
37	27000000	73	0.168	0.167	0.173	0.151	0.006	0.016
37	28000000	76	0.193	0.183	0.174	0.208	0.019	0.025
37	29000000	81	0.157	0.153	0.158	0.174	0.001	0.021
37	30000000	68	0.126	0.137	0.141	0.154	0.015	0.018
38	0	61	0.096	0.089	0.156	0.169	0.060	0.080
38	1000000	68	0.143	0.165	0.121	0.153	0.023	0.012
38	2000000	76	0.132	0.149	0.112	0.124	0.020	0.026
38	3000000	59	0.106	0.091	0.177	0.159	0.071	0.068
38	4000000	65	0.172	0.173	0.156	0.183	0.016	0.011
38	5000000	67	0.130	0.147	0.157	0.142	0.027	0.005
38	6000000	63	0.164	0.205	0.183	0.206	0.019	0.001
38	7000000	64	0.183	0.177	0.130	0.167	0.053	0.011
38	8000000	71	0.141	0.135	0.173	0.168	0.032	0.032
38	9000000	74	0.148	0.140	0.168	0.159	0.020	0.019
38	10000000	76	0.160	0.128	0.151	0.165	0.009	0.037
38	11000000	79	0.176	0.182	0.181	0.191	0.006	0.009
38	12000000	73	0.184	0.167	0.186	0.193	0.001	0.026
38	13000000	76	0.198	0.185	0.212	0.158	0.014	0.027
38	14000000	79	0.129	0.115	0.185	0.169	0.056	0.054
38	15000000	85	0.179	0.163	0.161	0.182	0.018	0.019
38	16000000	75	0.131	0.121	0.165	0.183	0.035	0.062
38	17000000	94	0.211	0.168	0.159	0.166	0.053	0.002
38	18000000	86	0.171	0.138	0.146	0.140	0.025	0.002
38	19000000	82	0.139	0.127	0.137	0.119	0.002	0.008
38	20000000	81	0.115	0.130	0.173	0.187	0.058	0.057
38	21000000	65	0.124	0.138	0.133	0.140	0.009	0.002
38	22000000	89	0.124	0.128	0.111	0.101	0.013	0.027
38	23000000	63	0.164	0.171	0.116	0.109	0.048	0.063
39	0	72	0.102	0.085	0.162	0.166	0.061	0.081
39	1000000	81	0.136	0.120	0.189	0.188	0.054	0.068
39	2000000	84	0.164	0.140	0.156	0.198	0.007	0.059
39	3000000	82	0.161	0.142	0.184	0.188	0.023	0.046
39	4000000	96	0.194	0.152	0.181	0.192	0.012	0.040
39	5000000	68	0.177	0.134	0.165	0.147	0.012	0.013
39	6000000	65	0.171	0.145	0.162	0.186	0.009	0.041
39	7000000	59	0.170	0.125	0.060	0.064	0.111	0.061
39	8000000	73	0.175	0.117	0.159	0.155	0.016	0.038
39	9000000	71	0.172	0.163	0.134	0.144	0.038	0.020
39	10000000	65	0.160	0.138	0.162	0.136	0.002	0.002
39	11000000	75	0.196	0.167	0.127	0.087	0.069	0.080
39	12000000	70	0.110	0.120	0.134	0.111	0.024	0.009
39	13000000	51	0.103	0.086	0.187	0.136	0.084	0.050
39	14000000	37	0.136	0.121	0.121	0.079	0.016	0.042
39	15000000	60	0.097	0.093	0.062	0.057	0.036	0.036
39	16000000	42	0.048	0.075	0.208	0.193	0.161	0.118
39	17000000	50	0.147	0.137	0.117	0.100	0.030	0.037
39	18000000	54	0.096	0.079	0.126	0.148	0.030	0.069
39	19000000	34	0.085	0.113	0.111	0.128	0.025	0.016
39	20000000	34	0.075	0.082	0.060	0.075	0.015	0.007
39	21000000	47	0.081	0.103	0.198	0.204	0.117	0.101
39	22000000	37	0.175	0.204	0.104	0.117	0.071	0.087
39	23000000	49	0.062	0.125	0.145	0.162	0.083	0.037
39	24000000	62	0.134	0.191	0.116	0.132	0.018	0.059
39	25000000	58	0.100	0.117	0.132	0.132	0.032	0.015
39	26000000	62	0.131	0.149	0.153	0.151	0.023	0.002
39	27000000	60	0.116	0.153	0.193	0.191	0.077	0.038
39	28000000	64	0.235	0.175	0.131	0.134	0.104	0.041
39	29000000	48	0.200	0.189	0.143	0.162	0.058	0.026
39	30000000	56	0.151	0.161	0.092	0.127	0.059	0.034
39	31000000	41	0.142	0.158	0.135	0.132	0.007	0.026
39	32000000	47	0.150	0.170	0.140	0.131	0.010	0.039
39	33000000	52	0.178	0.172	0.152	0.185	0.026	0.013
39	34000000	47	0.190	0.165	0.117	0.123	0.074	0.042
39	35000000	21	0.049	0.048	0.038	0.038	0.010	0.010
39	36000000	52	0.007	0.006	0.030	0.058	0.023	0.052
39	37000000	37	0.000	0.000	0.112	0.082	0.112	0.082
39	38000000	33	0.053	0.034	0.101	0.109	0.048	0.076
39	39000000	28	0.069	0.042	0.035	0.030	0.034	0.012
39	40000000	31	0.056	0.042	0.095	0.152	0.039	0.110
39	41000000	40	0.135	0.108	0.125	0.163	0.010	0.055
39	42000000	36	0.151	0.116	0.059	0.011	0.092	0.105
39	43000000	22	0.078	0.065	0.024	0.030	0.054	0.035
39	44000000	19	0.011	0.011	0.004	0.006	0.007	0.005
39	45000000	20	0.000	0.007	0.000	0.009	0.000	0.002
39	46000000	17	0.000	0.000	0.028	0.027	0.028	0.027
39	47000000	15	0.036	0.038	0.036	0.059	0.000	0.022
39	48000000	7	0.080	0.081	0.000	0.000	0.080	0.081
39	49000000	15	0.014	0.014	0.029	0.031	0.015	0.017
39	50000000	14	0.030	0.016	0.004	0.007	0.026	0.009
39	51000000	19	0.011	0.011	0.006	0.003	0.005	0.008
39	52000000	15	0.021	0.018	0.000	0.000	0.021	0.018
39	53000000	26	0.008	0.008	0.022	0.023	0.014	0.015
39	54000000	28	0.046	0.046	0.023	0.013	0.023	0.033
39	55000000	37	0.037	0.035	0.056	0.033	0.019	0.002
39	56000000	25	0.038	0.054	0.010	0.033	0.028	0.022
39	57000000	25	0.034	0.033	0.017	0.023	0.018	0.009
39	58000000	15	0.044	0.038	0.028	0.040	0.017	0.002
39	59000000	35	0.062	0.068	0.164	0.236	0.102	0.168
39	60000000	28	0.253	0.215	0.031	0.102	0.222	0.113
39	61000000	24	0.093	0.085	0.053	0.178	0.040	0.092
39	62000000	33	0.142	0.136	0.034	0.058	0.108	0.078
39	63000000	33	0.000	0.003	0.056	0.089	0.056	0.086
39	64000000	18	0.000	0.000	0.169	0.199	0.169	0.199
39	65000000	20	0.038	0.053	0.026	0.078	0.012	0.025

39	66000000	21	0.036	0.049	0.024	0.040	0.012	0.010
39	67000000	38	0.053	0.069	0.043	0.104	0.010	0.035
39	68000000	34	0.083	0.086	0.082	0.130	0.001	0.043
39	69000000	43	0.063	0.076	0.132	0.193	0.069	0.117
39	70000000	51	0.115	0.125	0.046	0.064	0.069	0.061
39	71000000	30	0.114	0.104	0.157	0.215	0.044	0.111
39	72000000	24	0.231	0.128	0.157	0.149	0.074	0.021
39	73000000	33	0.101	0.081	0.141	0.101	0.040	0.020
39	74000000	52	0.085	0.065	0.096	0.085	0.011	0.019
39	75000000	40	0.089	0.070	0.065	0.051	0.024	0.019
39	76000000	26	0.063	0.055	0.016	0.027	0.047	0.027
39	77000000	25	0.023	0.021	0.017	0.032	0.005	0.011
39	78000000	44	0.000	0.000	0.063	0.071	0.063	0.071
39	79000000	43	0.027	0.021	0.029	0.023	0.002	0.001
39	80000000	30	0.013	0.008	0.005	0.008	0.007	0.000
39	81000000	34	0.006	0.005	0.030	0.025	0.024	0.020
39	82000000	32	0.001	0.001	0.054	0.067	0.053	0.066
39	83000000	35	0.004	0.003	0.035	0.056	0.031	0.053
39	84000000	55	0.001	0.001	0.013	0.053	0.012	0.052
39	85000000	64	0.000	0.000	0.019	0.033	0.019	0.033
39	86000000	35	0.023	0.019	0.108	0.079	0.086	0.060
39	87000000	38	0.089	0.079	0.049	0.075	0.040	0.003
39	88000000	55	0.065	0.052	0.003	0.011	0.062	0.041
39	89000000	26	0.008	0.007	0.027	0.060	0.019	0.053
39	90000000	47	0.042	0.030	0.105	0.141	0.063	0.111
39	91000000	49	0.137	0.137	0.114	0.158	0.023	0.021
39	92000000	38	0.148	0.161	0.106	0.092	0.042	0.068
39	93000000	32	0.121	0.147	0.153	0.194	0.032	0.047
39	94000000	67	0.222	0.237	0.138	0.166	0.083	0.071
39	95000000	49	0.155	0.163	0.134	0.148	0.020	0.015
39	96000000	59	0.135	0.157	0.083	0.101	0.052	0.056
39	97000000	45	0.041	0.022	0.017	0.168	0.024	0.147
39	98000000	39	0.013	0.013	0.146	0.178	0.134	0.165
39	99000000	47	0.066	0.040	0.178	0.227	0.113	0.186
39	100000000	59	0.193	0.156	0.148	0.227	0.045	0.071
39	101000000	52	0.153	0.171	0.026	0.213	0.127	0.042
39	102000000	46	0.134	0.230	0.232	0.318	0.098	0.088
39	103000000	57	0.298	0.316	0.163	0.212	0.135	0.104
39	104000000	45	0.201	0.223	0.055	0.094	0.146	0.130
39	105000000	33	0.099	0.139	0.027	0.058	0.072	0.080
39	106000000	45	0.059	0.073	0.213	0.367	0.154	0.294
39	107000000	32	0.184	0.349	0.054	0.094	0.130	0.255
39	108000000	35	0.072	0.095	0.057	0.100	0.014	0.005
39	109000000	34	0.098	0.082	0.099	0.126	0.001	0.044
39	110000000	48	0.124	0.176	0.101	0.158	0.023	0.018
39	111000000	59	0.164	0.205	0.168	0.166	0.004	0.039
39	112000000	62	0.137	0.157	0.077	0.090	0.060	0.067
39	113000000	50	0.103	0.095	0.059	0.096	0.043	0.001
39	114000000	49	0.064	0.080	0.074	0.082	0.010	0.001
39	115000000	39	0.101	0.124	0.047	0.046	0.055	0.078
39	116000000	29	0.043	0.087	0.101	0.080	0.058	0.007
39	117000000	23	0.084	0.129	0.183	0.193	0.099	0.064
39	118000000	28	0.133	0.145	0.085	0.105	0.048	0.040
39	119000000	29	0.068	0.100	0.005	0.021	0.063	0.079
39	120000000	17	0.056	0.073	0.056	0.054	0.001	0.020
39	121000000	35	0.116	0.140	0.127	0.073	0.011	0.067
39	122000000	34	0.111	0.092	0.046	0.049	0.065	0.043
